# Ultra-low-dose CT vs. chest X-ray in non-traumatic emergency department patients – a prospective randomised crossover cohort trial

**DOI:** 10.1016/j.eclinm.2023.102267

**Published:** 2023-10-17

**Authors:** Christian Wassipaul, Karin Janata-Schwatczek, Hans Domanovits, Dietmar Tamandl, Helmut Prosch, Martina Scharitzer, Stephan Polanec, Ruediger E. Schernthaner, Thomas Mang, Ulrika Asenbaum, Paul Apfaltrer, Filippo Cacioppo, Nikola Schuetz, Michael Weber, Peter Homolka, Wolfgang Birkfellner, Christian Herold, Helmut Ringl

**Affiliations:** a Department of Biomedical Imaging and Image-guided Therapy, Medical University of Vienna, Austria; b Department of Emergency Medicine, Medical University of Vienna, Austria; c Diagnostikum Graz, Austria; d Department of Diagnostic and Interventional Radiology, Clinic Landstrasse, Vienna Healthcare Group, Austria; e Department of Radiology, Medical University of Graz, Austria; f Centre for Medical Physics and Biomedical Engineering, Medical University of Vienna, Austria; g Department of Diagnostic and Interventional Radiology, Clinic Donaustadt, Vienna Healthcare Group, Austria

**Keywords:** Ultra-low-dose computed tomography, Chest X-ray, Non-traumatic emergency department, Detection rate, Primary imaging modality

## Abstract

**Background:**

Ultra-low-dose CT (ULDCT) examinations of the chest at only twice the radiation dose of a chest X-ray (CXR) now offer a valuable imaging alternative to CXR. This trial prospectively compares ULDCT and CXR for the detection rate of diagnoses and their clinical relevance in a low-prevalence cohort of non-traumatic emergency department patients.

**Methods:**

In this prospective crossover cohort trial, 294 non-traumatic emergency department patients with a clinically indicated CXR were included between May 2nd and November 26th of 2019 (www.clinicaltrials.gov: NCT03922516). All participants received both CXR and ULDCT, and were randomized into two arms with inverse reporting order. The detection rate of CXR was calculated from ‘arm CXR’ (n = 147; CXR first), and of ULDCT from ‘arm ULDCT’ (n = 147; ULDCT first). Additional information reported by the second exam in each arm was documented. From all available clinical and imaging data, expert radiologists and emergency physicians built a compound reference standard, including radiologically undetectable diagnoses, and assigned each finding to one of five clinical relevance categories for the respective patient.

**Findings:**

Detection rates for main diagnoses by CXR and ULDCT (mean effective dose: 0.22 mSv) were 9.1% (CI [5.2, 15.5]; 11/121) and 20.1% (CI [14.2, 27.7]; 27/134; *P* = 0.016), respectively. As an additional imaging modality, ULDCT added 9.1% (CI [5.2, 15.5]; 11/121) of main diagnoses to prior CXRs, whereas CXRs did not add a single main diagnosis (0/134; *P* < 0.001). Notably, ULDCT also offered higher detection rates than CXR for all other clinical relevance categories, including findings clinically irrelevant for the respective emergency department visit with 78.5% (CI [74.0, 82.5]; 278/354) vs. 16.2% (CI [12.7, 20.3]; 58/359) as a primary modality and 68.2% (CI [63.3, 72.8]; 245/359) vs. 2.5% (CI [1.3, 4.7]; 9/354) as an additional imaging modality.

**Interpretation:**

In non-traumatic emergency department patients, ULDCT of the chest offered more than twice the detection rate for main diagnoses compared to CXR.

**Funding:**

The Department of Biomedical Imaging and Image-guided Therapy of Medical University of Vienna received funding from 10.13039/501100011699Siemens Healthineers (Erlangen, Germany) to employ two research assistants for one year.

**Research in context**

**Evidence before this study**

The PubMed database was searched for studies published in English on ultra-low-dose CT (ULDCT) of the chest published through May of 2023. The search term “(‘ultra-low-dose’ OR ‘low-dose’) AND (‘computed tomography’ OR ‘CT’) AND (‘chest’ OR ‘thorax’ OR ‘thoracic')” gave 2695 results, while the addition of “AND (‘CXR’ OR ‘chest X-ray’ OR ‘radiography’)” narrowed it to 2129 results, and the further addition of “AND ‘emergency'” resulted in 94 studies. The search term “(‘effective dose’ OR ‘effective dose conversion coefficient’ OR ‘radiation dose’) AND (‘ultra-low-dose’ OR ‘low-dose’) AND (‘computed tomography OR ‘CT')” yielded 7158 results and the addition of the term “AND ‘cancer risk computed tomography’” narrowed this to 1072 studies. We identified 259 studies of interest (published between January 1999 and May 2023). Among these, we identified four studies that assessed ULDCT at a similar dose level compared to CXR, but with different study questions and setups, which we have addressed in detail in the Discussion section. To the best of our knowledge, there are currently no studies evaluating thoracic ULDCT and its intra-patient clinical relevance as a primary imaging alternative to CXR in non-traumatic emergency department patients without limitations on the spectrum of indications constraints on body mass index (BMI). Hence, evidence concerning the clinical relevance of ULDCT of the chest versus CXR in a non-traumatic patient cohort of an emergency department is scarce.

**Added value of this study**

To the best of our knowledge, this is the first study to conduct an intra-patient comparison of ULDCT of the chest and CXR with regard to their clinical relevance in a non-traumatic patient cohort of an emergency department without limitations on the spectrum of indications or constraints on BMI (n = 294). In this study cohort, ULDCT of the chest offered more than twice the detection rate for main diagnoses compared to chest X-ray.

**Implications of all the available evidence**

Resources provided, this trial supports the future use of ULDCT as one of the primary chest-imaging modalities in low-prevalence non-traumatic emergency department patients. Due to higher expected costs and resource requirements, further economic analyses will be necessary to determine the optimal spectrum of indications, rationale and economic potential of a broader substitution of ULDCT for CXR as a primary imaging modality of the chest.

## Introduction

Non-traumatic patients accounted for approximately 99 million annual emergency department (ED) visits in the USA in 2017. Based on estimates from the National Hospital Ambulatory Medical Care Survey (NHAMCS), 12.5 million of these received a chest X-ray (CXR) and 2.0 million a computed tomography (CT) scan of the chest.^1^

Its low cost, widespread availability, short reporting time, and low radiation exposure (mean effective dose of a CXR in two views of 0.1 mSv^2^, ^3^, ^4^) make CXR an excellent primary diagnostic test for a broad range of indications, with a low threshold for referral.^5^, ^6^, ^7^, ^8^ However, its sensitivity and specificity are moderate, mainly due to superposition of anatomic structures.^9^^,^^10^ In contrast, chest CT offers a three-dimensional (3D) image acquisition without superposition, but with the disadvantages of a markedly higher radiation dose, higher costs, as well as longer examination and reporting times.^11^ Therefore, in non-traumatic emergency medicine, CT as a primary imaging modality is typically limited to specific symptom constellations in high-prevalence patient groups, or used as a secondary imaging modality, e.g., after inconclusive CXR.^12^ However, the rule-out of pneumonia using chest CT in patients suspected of non-traumatic pulmonary disease in the ED is emerging as a promising technique.^13^, ^14^, ^15^, ^16^

In current clinical routine, non-contrast-enhanced chest CTs are performed at two major radiation dose levels, standard-dose CT (SDCT; 6 mSv^3^) and low-dose CT (LDCT; 1.2 to 2 mSv^16^, ^17^, ^18^). However, the latest advances in CT technology, including new detectors and new reconstruction algorithms, have considerably lowered radiation exposure requirements for chest CTs to nearly the radiation dose of a CXR.^19^, ^20^, ^21^, ^22^ Thus, ultra-low-dose CT (ULDCT; 0.22 mSv^17^) of the chest is emerging as a diagnostic alternative to conventional CXR in low-prevalence patient groups, and several studies have shown the feasibility of chest CTs for specific indications at a radiation dose of up to three chest X-ray examinations (0.05–0.3 mSv).^17^^,^^23^, ^24^, ^25^, ^26^, ^27^, ^28^ However, to the best of the authors' knowledge, there are currently no studies that have evaluated thoracic ULDCT and its intra-patient clinical relevance as a primary imaging alternative to CXR in a low-prevalence cohort of non-traumatic ED patients, without limiting the spectrum of indications or BMI.

We hypothesize that the use of thoracic ULDCT as a primary imaging alternative to CXR in a low-prevalence cohort of non-traumatic ED patients, without limitations on the spectrum of indications or constraints on BMI, offers a higher detection rate for all assessed clinical relevance categories.

Therefore, the aim of this trial was to prospectively compare ULDCT and CXR for the detection rate of diagnoses and their clinical relevance in a low-prevalence non-traumatic ED patient cohort.

## Methods

### Trial participants and recruitment

In this trial, non-traumatic emergency department (ED) patients from our institution, with a clinically indicated CXR were included, if written, informed consent was provided. The trial protocol was approved by the local institutional review board (No. 2254/2018) and registered at www.clinicaltrials.gov (NCT03922516), with the acronym UP-Chest trial (ULDCT versus Plain film of the Chest). Patients whose critical condition prohibited two imaging examinations and pregnant women were not eligible for recruitment. Women younger than 55 years were tested for β-HCG. Patient recruitment was carried out daily during the hours of 8.30 am to 2 pm up to a maximum of six patients per day, limited by the available resources at the dedicated CT scanner in the full clinical routine. Within these limits, inclusion was done sequentially. Inclusion and exclusion criteria are summarized in Supplement Table E1.

### Trial design

**Fig. 1:**
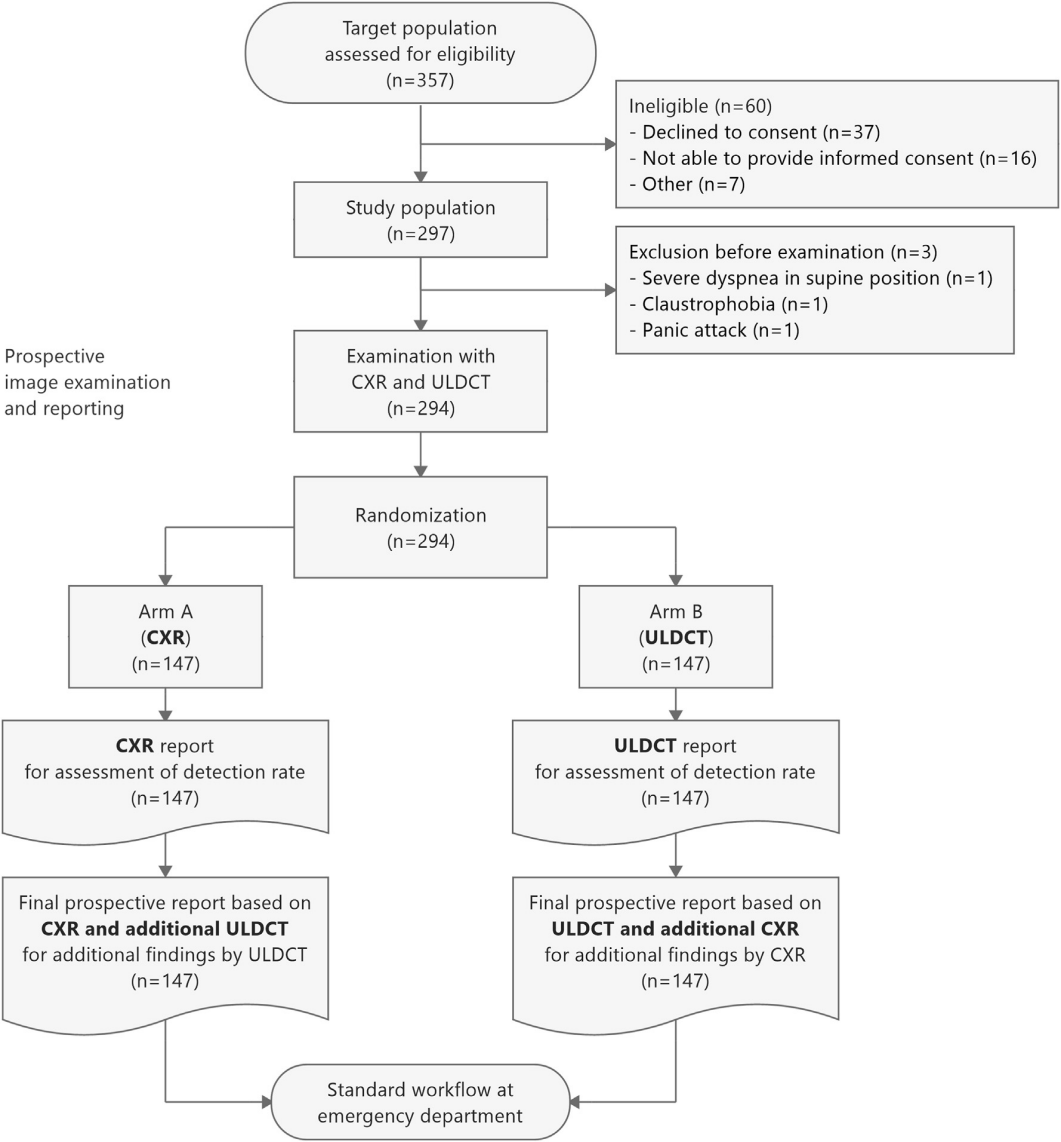
**Prospective trial workflow within the clinical routine**. Using only the first imaging modality of the respective arm for the determination of detection rate of CXR and ULDCT, this setup was chosen to exclude an intra-reader bias.

The entry point for all patients of this prospective crossover cohort trial was the clinical indication for a CXR. Trial participants were sequentially examined with both imaging methods, CXR and ULDCT of the chest as an intervention, and then alternately randomized into two arms with an inverse reporting order. For patients in arm A (‘arm CXR’), the attending radiology consultant first filed the report on the CXR and only this report was used to determine the detection rate of CXR to avoid an intra-reader bias (Fig. 1). Immediately afterward, the same radiologist read the ULDCT images and filed an additional report based on the imaging data of both examinations to reach the clinically required final intra-reader consensus for that patient. This consensus report was not used for the detection rate of CXR, but only to investigate whether ULDCT could add any information to a prior CXR report. In arm B (‘arm ULDCT’), the reporting order was reversed, and the radiology consultant first filed the report on the ULDCT, which was used to assess its detection rate. The subsequent combined report on both imaging examinations was used to determine additional findings of CXR to a prior ULDCT.

All radiological reports were written within the standard clinical workflow at our institution, which included routine access to prior patient history and imaging data. Image reading was performed using the local picture archiving and communication system equipment. Enrolment, allocation to trial arms, and documentation were conducted by the trial coordinator (C.W.).

#### Compound reference standard and clinical relevance

All prospective imaging reports were transformed into dichotomous radiological report data after the elimination of synonyms, to enable binary comparison for further assessment.

**Fig. 2:**
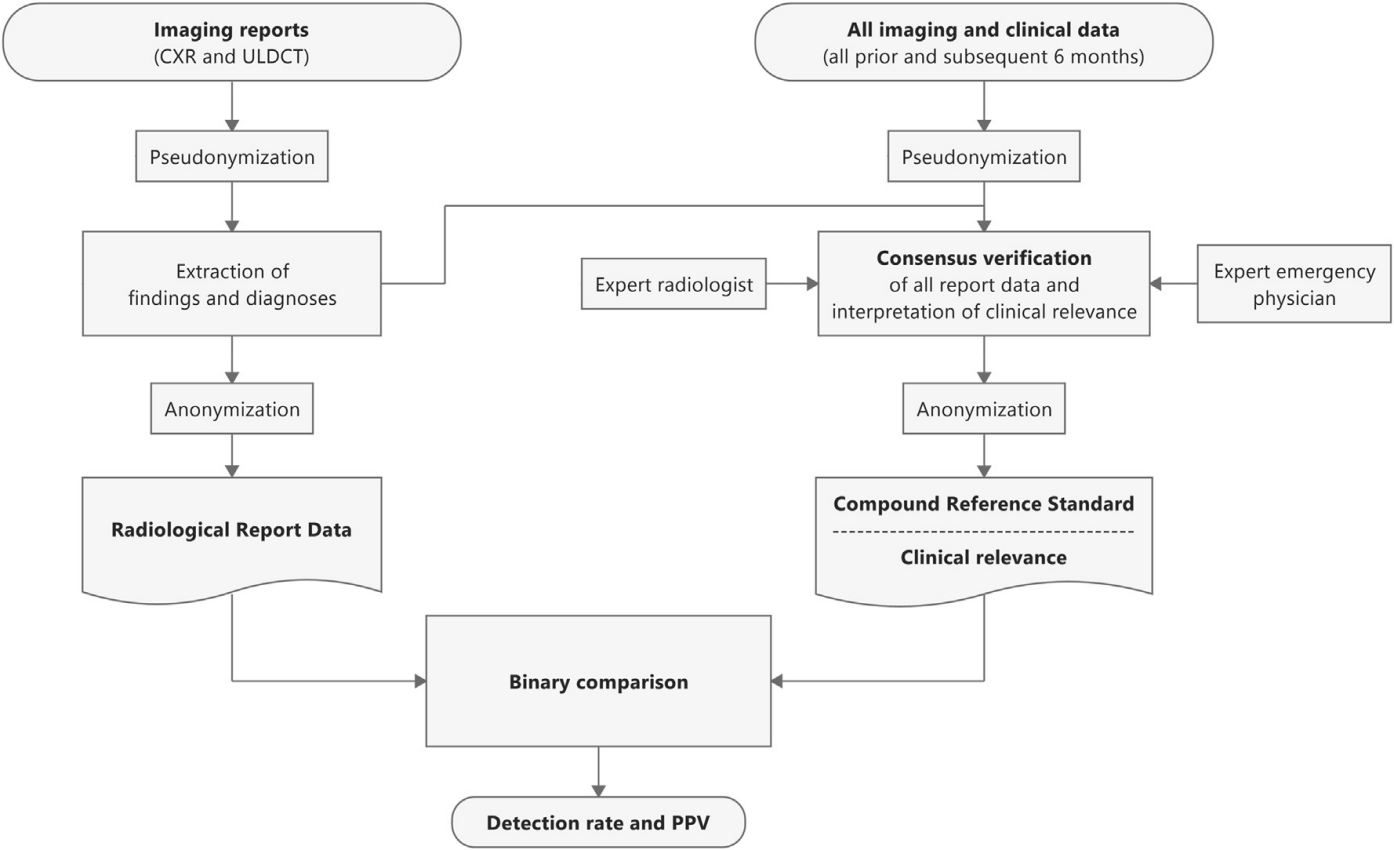
**Image analysis and building of a Compound Reference Standard (CompRS)**.

Building of the compound reference standard (CompRS) was a complex, inter-disciplinary, multi-level process, visualized in Fig. 2 and provided in detail in the Supplements. Expert CT radiologists and expert emergency physicians assessed all imaging findings and clinical diagnoses and considered all other available imaging and clinical data up to six months after the end of recruitment to reach a conclusive consensus set of validated imaging findings and diagnoses for every patients initial emergency department visit. Imaging and clinical data after the initial emergency department visit were assessed only to validate or falsify the initial diagnoses in the case of findings and diagnoses that can only be verified over time, such as pulmonary nodules in contrast to acute inflammation. Notably, this CompRS contained radiological findings, as well as clinical diagnoses, including those undetectable by imaging methods. All CompRS elements were stratified with regard to their individual clinical relevance for every patient within the respective hospital visit. The rationale for these clinical relevance categories - similar to Goldman et al. but adapted to the clinical requirements of this trial - is explained in detail in the Supplementary Material.^29^ The categories ranged from the ‘main diagnosis’ that caused this ED visit, to ‘important incidental diagnosis,’ ‘strongly contributing,’ ‘moderately contributing,’ and ‘clinically irrelevant findings’ for this ED visit. Within this trial, the same diagnosis or finding might be classified as a different clinical relevance category in different patients, since in clinical routine the relevance of a finding/diagnosis depends on the patients' individual reason for the specific emergency department visit. Two such examples are given in the Supplementary Material. However, the clinical relevance categories were mutually exclusive within each individual patient. To avoid overemphasis of exclusions of main referring diagnoses, such as ‘no signs of pneumothorax,’ rule-outs were not classified as a ‘main diagnosis,’ but only as a ‘strongly contributing finding’ or ‘moderately contributing finding’. However, since the exclusion of frequent thoracic pathologies is one of the main purposes of CXR in emergency medicine, rule-outs of main referring diagnoses were also analysed.

#### Outcome measures

To assess both imaging methods with regard to clinical relevance, the detection rate for the clinical relevance categories of prospective reports and the proportion of correct results (positive predictive value, PPV) of CXR was calculated from ‘arm CXR’ (first modality: CXR) and of ULDCT from ‘arm ULDCT’ (first modality: ULDCT). For both imaging modalities, as a primary and additional imaging modality, the PPV was calculated by comparing the findings of the respective imaging modality against the CompRS. Sensitivity and specificity were not applied, since they are reserved for tests of specific diseases rather than groups of diseases. While the term ‘sensitivity’ itself it is not applicable to the clinical relevance categories used in our trial, the term ‘detection rate’ does not contain this limitation. Mathematically, both terms are computed identically.

As a result of the transformation of imaging reports and the CompRS into dichotomous data, for each patient, our database contains a 0 or a 1 for all 181 different findings observed in this trial. Results were calculated via binary comparison of dichotomous prospective report data against the CompRS for the individual patient.

As is common in clinical routine, a large proportion of ED main diagnoses were not directly visualizable by either ULDCT or CXR (e.g., musculoskeletal thoracic pain or atrial fibrillation). The results, therefore, do not directly reflect the radiological ability of CXR and ULDCT to visualize the respective diseases, but rather their clinical contribution to the final diagnoses.

### Changes in patient management

Immediately after reading the report of each imaging examination in this trial, the treating emergency physician was asked to indicate whether the respective examination caused any change in management. Results were acquired separately from each other for the primary and additional imaging modality of both trial arms.

### Image acquisition and radiation dose

All CXR examinations were performed with the standard digital chest radiography system at our institution using standard imaging parameters. Depending on mobility and clinical condition, patients were examined in one or two views. ULDCTs were acquired on a third-generation 2 × 128-row Multi-Detector Dual-Source CT System (Somatom Drive, Siemens Healthineers, Erlangen, Germany) using a non-contrast-enhanced CT protocol with 100 kV, tin-filtration, and 50 ref-mAs. Device specifications and imaging protocol details are provided in the Supplements (Tables E2-E3).

In the European Union and USA, a CXR examination in two views requires a mean effective radiation dose of 0.1 mSv and is further referred to as a ‘standard CXR examination.’^2^, ^3^, ^4^ The targeted effective radiation dose of 0.2 mSv for a ULDCT in this trial corresponds to twice the mean effective radiation dose of such a standard CXR examination. Details for the calculation of radiation dose estimates are given in the Supplements.

### Statistical analysis

All statistical computations were performed by a biostatistician (M.W.) using IBM SPSS Statistics version 27.0 (© IBM, Armonk, NY). The required number of patients was calculated via power analysis (Supplements).

To compare the two trial arms, unpaired student t-tests were used for metric and normally distributed data. In case of skewed metric data, Mann-Whitney-U-tests were used. A chi^2^ test was used to compare the sex distribution of the two arms. To compare percentages based on diagnoses (e.g., detections rates or PPV), Generalized Estimation Equations (GEE) were used to consider multiple diagnoses per patient.

Metric data are described using means ± SD. For nominal data, absolute frequencies and percentages are used. A *P*-value equal to or below 0.05 was considered statistically significant.

### Role of the funding source

The Department of Biomedical Imaging and Image-guided Therapy of Medical University of Vienna received funding from Siemens Healthineers (Erlangen, Germany) to employ two research assistants for one year. Limited participation of the funding source in the adaptation of the technical aspects of the applied CT protocols is disclosed. Throughout the trial, all data exclusively remained under the control of the Medical University of Vienna and was never disclosed to third parties. Third parties did not participate in and had no influence on data acquisition, analysis, the conduct, or reporting of this trial.

## Results

### Patient recruitment and characteristics

Up to six patients per day were recruited from May 2nd to November 26th of 2019. In total, 357 patients were eligible, of which 297 patients were included in this trial. A preliminary analysis revealed a lower drop-out rate than assumed, ensuring the required power for this trial, and therefore, patient inclusion was stopped. Three patients had to be excluded, resulting in a final cohort of 294 patients, 147 in each trial arm (Fig. 1 and Supplements Table E4). Demographic patient characteristics are provided in Table 1 and in the Supplements (Table E5).

**Table 1:** Patient demographics and symptoms at presentation.

Patient demographics	Total		*P*
	Arm CXR (CXR—ULDCT)	Arm ULDCT (ULDCT—CXR)	
No. of patients	294		–
	147	147	
Sex	86 male; 61 female	77 male; 70 female	0.291
Age (years, mean ± SD [range])	58.0 years ± 20.5 (18.9–92.6)		0.603
	58.6 ± 20.3 (20.4–92.6)	57.4 ± 20.7 (18.9–91.7)	
Height (cm, mean ± SD [range])	171.7 cm ± 10.2 (143–200)		0.300
	172.3 ± 10.7 (148–200)	171.1 ± 9.6 (143–198)	
Weight (kg, mean ± SD [range])	78.2 kg ± 18.2 (35–140)		0.167
	79.7 ± 18.5 (35–140)	76.7 ± 17.9 (45–135)	
BMI (kg/m^2^, mean ± SD [range])	26.5 kg/m^2^ ± 5.6 (13.1–47.8)		0.383
	26.8 ± 5.5 (13.7–46.1)	26.2 ± 5.8 (13.1–47.8)	
**Symptoms at presentation**	**Arm CXR (CXR—ULDCT)**	**Arm ULDCT (ULDCT—CXR)**	***P***

Thoracic pain	106/294 (36.1%)		0.145
	59/147 (40.1%)	47/147 (32.0%)	
Dyspnoea	77/294 (26.2%)		0.894
	39/147 (26.5%)	38/147 (25.9%)	
Cough	36/294 (12.2%)		0.722
	19/147 (12.9%)	17/147 (11.6%)	
Impaired general condition	25/294 (8.5%)		0.530
	11/147 (7.5%)	14/147 (9.5%)	
Fever	18/294 (6.1%)		0.627
	8/147 (5.4%)	10/147 (6.8%)	

### Findings and their clinical relevance

#### Compound reference standard

The Compound Reference Standard (CompRS, all imaging findings and clinical diagnoses) contained a total of 1580 elements, resulting in a mean of 5.4 elements per patient, which showed no significant difference (*P* = 0.746) between arm CXR (5.3 ± 2.7) and arm ULDCT (5.4 ± 3.0). These 1580 elements contained a total of 314 main diagnoses (including 255 identified main diagnoses and 59 patients for whom no main diagnosis as reason for the respective ED visit was found), 44 important incidental diagnoses, 67 strongly contributing findings, 442 moderately contributing findings, and 713 clinically irrelevant findings.

The most frequent main diagnoses for all patients were ‘no main diagnosis found’ (n = 59; arm A (CXR) n = 31; arm B (ULDCT) n = 28), ‘musculoskeletal thoracic pain’ (n = 36; arm A (CXR) n = 14; arm B(ULDCT) n = 22), ‘pneumonia’ (n = 31; arm A (CXR) n = 14; arm B (ULDCT) n = 17), and ‘cardiac decompensation’ (n = 23; arm A (CXR) n = 11; arm B (ULDCT) n = 12), of which imaging can directly detect only pneumonia (detailed results in the Supplements, Table E6). By convention in this trial, only such direct confirmations of the CompRS by imaging were considered true-positives. The main diagnosis ‘no main diagnosis found’ was, therefore, excluded from the detection rate calculation.

#### Reporting performance of CXR and ULDCT

**Fig. 3:**
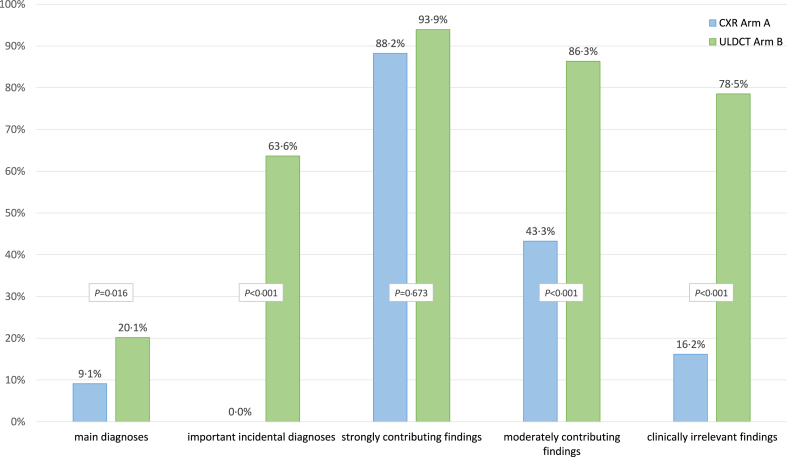
**Detection rate of primary CXR and primary ULDCT**. Proportion of ‘main diagnoses,’ ‘important incidental diagnoses,’ ‘strongly contributing findings,’ ‘moderately contributing findings,’ and ‘clinically irrelevant findings’ detected by CXR (in arm A) and ULDCT (in arm B) relative to the total number in the Compound Reference Standard of the respective arm. Notably, the Compound Reference Standard contained radiological findings, as well as clinical diagnoses, including those undetectable by imaging methods.

**Fig. 4:**
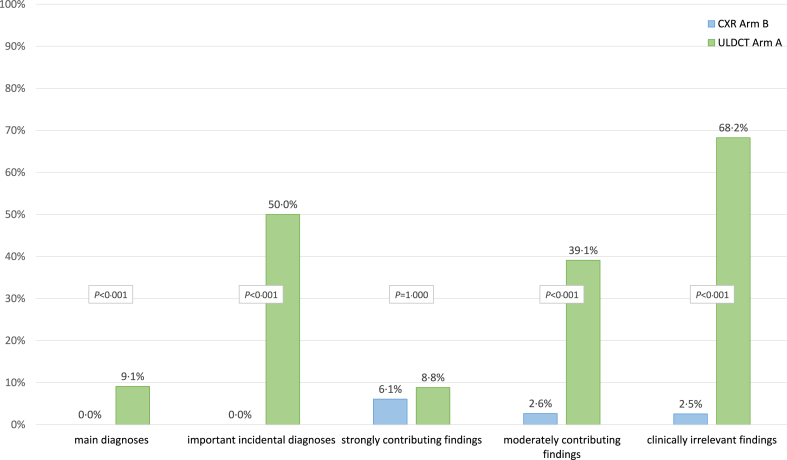
**Additional findings and diagnoses by CXR and ULDCT**. Proportion of ‘main diagnoses,’ ‘important incidental diagnoses,’ ‘strong contributing findings,’ ‘moderately contributing findings,’ and ‘clinically irrelevant findings’ added by CXR to prior ULDCT (in arm B) and by ULDCT to prior CXR (in arm A) relative to the total number in the Compound Reference Standard of the respective arm.

**Fig. 5:**
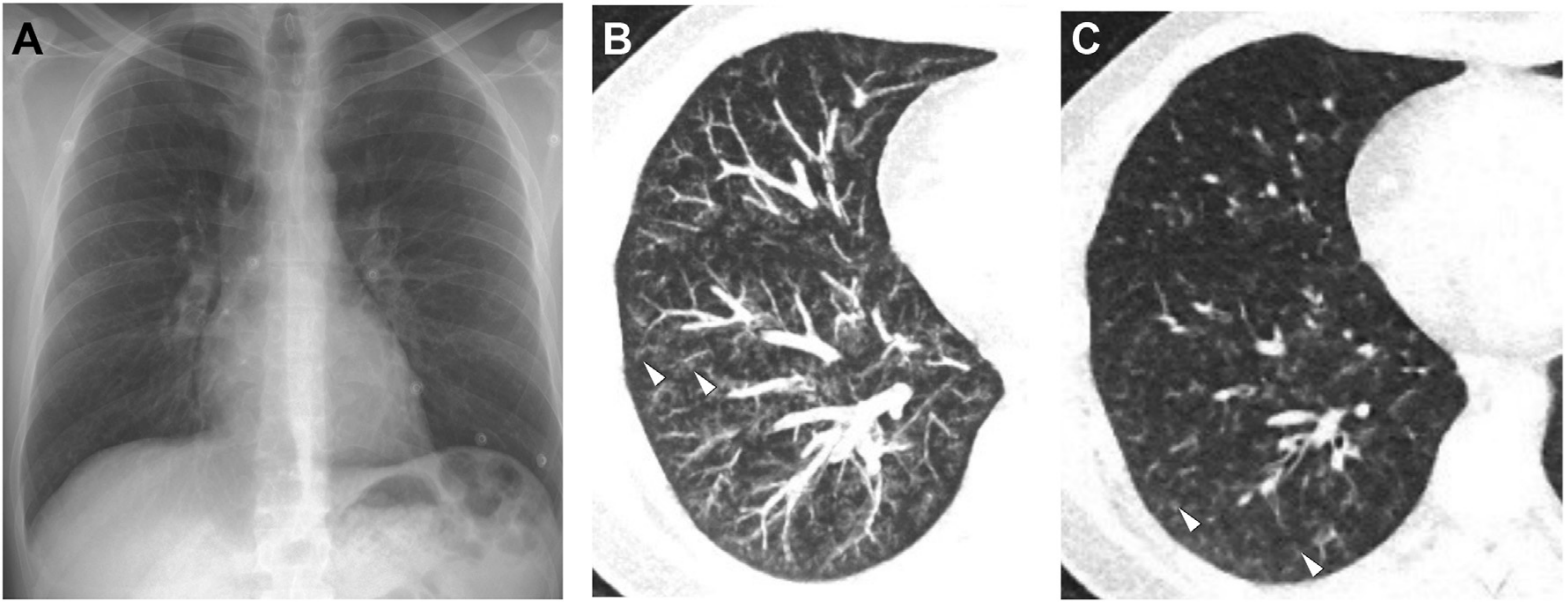
**Example A.** A 38-year-old male patient who presented with dyspnoea, cough, and fever of up to 39 °C for two weeks and no improvement under antibiotics. CXR showed moderate bronchial wall thickening (A), but ULDCT detected extensive bilateral centrilobular nodules (B, C—white arrowheads) with basal predominance, consistent with bronchiolitis.

**Fig. 6:**
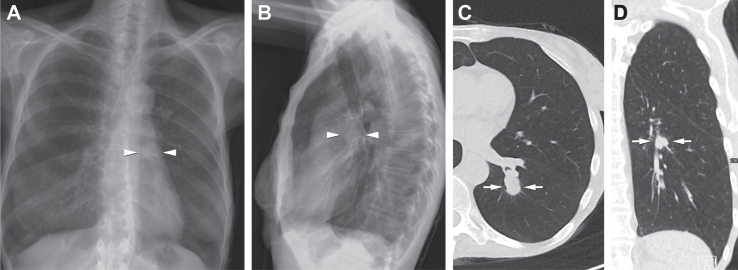
**Example B.** A 52-year-old female patient with a history of smoking, COPD, and prior oral cancer who presented with an impaired general condition and elevated CRP. The CXR report described only findings consistent with pulmonary emphysema. The following ULDCT additionally detected a pulmonary nodule suspicious for malignancy of the central left apical lower lobe (C, D—white arrows). Retrospectively, with the knowledge of the ULDCT, this nodule could be also suspected on CXR in superposition of the left pulmonary artery (A, B—white arrowheads).

As a first imaging modality, prospective ULDCT reports (Arm B) contained a significantly higher number of findings and diagnoses than CXR with a total of 574 vs. 257 (*P* < 0.001). Of these, 546 (ULDCT) and 192 (CXR) were verified in the CompRS (*P* < 0.001), resulting in a significantly higher positive predictive value of 95.1% CI [93.0, 96.6] for ULDCT vs. 74.7% CI [69.1, 79.6] for CXR (*P* < 0.001). With regard to clinical relevance, ULDCT provided a significantly higher detection rate for ‘main diagnoses,’ with 20.1% (CI [14.2, 27.7]; 27/134 confirmed main diagnoses in arm ULDCT), compared to 9.1% by CXR (CI [5.2, 15.5]; 11/121; *P* = 0.016). The corresponding results for ‘important incidental diagnoses’ were 63.6% (CI [43.0, 80.3]; 14/22) for ULDCT and 0.0% (CI [0.0, 14.9]; 0/22) for CXR (*P* < 0.001), as well as 86.3% (CI [81.3, 90.2]; 196/227) and 43.3% (CI [36.8, 49.9]; 93/215) for ‘moderately contributing findings’ (*P* < 0.001), respectively. The proportion of ‘strongly contributing findings’ detected by ULDCT (93.9%; CI [80.4, 98.3]; 31/33) and CXR (88.2%; CI [73.4, 95.3]; 30/34) in the respective arms did not significantly differ (*P* = 0.673), most likely because of the high proportion of rule-outs in this group, indicating the importance of rule-outs of frequent pathologies by CXR in ED patients. Detailed results are shown in Table 2, Table 3, Fig. 3, Fig. 4 and in the Supplements (Table E6). Examples are provided in Fig. 5, Fig. 6 and in the Supplements (Figures E1-E3).

**Table 2:** Diagnoses and findings directly detected by CXR and ULDCT in relation to the Compound Reference Standard.

Clinical categories detected by primary imaging, including radiologically undetectable diagnoses	Arm CXR	Arm ULDCT	*P*
	CXR detected	ULDCT detected	
Main diagnoses (other than “no main diagnosis found”)	11/121 (9.1%)	27/134 (20.1%)	0.016
Important incidental diagnoses	0/22 (0.0%)	14/22 (63.6%)	<0.001
Strongly contributing findings	30/34 (88.2%)	31/33 (93.9%)	0.673
Moderately contributing findings	93/215 (43.3%)	196/227 (86.3%)	<0.001
Clinically irrelevant findings	58/359 (16.2%)	278/354 (78.5%)	<0.001
**Clinical categories detected by additional imaging, including radiologically undetectable diagnoses**	**Arm CXR**	**Arm ULDCT**	***P***
	ULDCT added	CXR added	

Main diagnoses (other than “no main diagnosis found”)	11/121 (9.1%)	0/134 (0.0%)	<0.001
Important incidental diagnoses	11/22 (50.0%)	0/22 (0.0%)	<0.001
Strongly contributing findings	3/34 (8.8%)	2/33 (6.1%)	1.000
Moderately contributing findings	84/215 (39.1%)	6/227 (2.6%)	<0.001
Clinically irrelevant findings	245/359 (68.2%)	9/354 (2.5%)	<0.001
**Exclusions of referring diagnosis**	**Arm CXR**	**Arm ULDCT**	***P***
	by CXR	by ULDCT	

Total (exclusions of referring diagnosis/patients per arm)	103/147	112/147	0.236
Correct	94/103 (91.3%)	109/112 (97.3%)	0.053
Strongly contributing findings	25/94 (26.6%)	24/109 (22.0%)	0.741
Moderately contributing findings	68/94 (72.3%)	84/109 (77.1%)	
Clinically irrelevant findings	1/94 (1.1%)	1/109 (0.9%)	
False	9/103 (8.7%)	3/112 (2.7%)	0.053

Notably, the Compound Reference Standard contained radiological findings, as well as clinical diagnoses, including those undetectable by imaging methods.

We emphasise, that “no main diagnosis found” (n = 59) is not included in this table.

**Table 3:** Detection rate of CXR and ULDCT as primary imaging modality (CXR in ‘Arm CXR’ and ULDCT in ‘Arm ULDCT’).

Arm CXR: Detection rate of CXR - findings and diagnoses stratified according to clinical relevance										
Rank	Main diagnoses	11	Important incidental diagnoses	0	Strongly contributing findings	30	Moderately contributing findings	93	Clinically irrelevant findings^a^	58
1	Pneumonia	9	–	–	Excl. of pneumonia	10	Excl. of pneumonia	24	Aortic atherosclerosis	32
2	Pleural effusion	1	–	–	Excl. of pneumothorax	9	Excl. of pulm. edema	16	Enlarged heart	4
3	Dynamic of known lung cancer	1	–	–	Excl. of pulm. edema	3	Excl. of pneumothorax	11	Pleural thickening	4
4	–	–	–	–	Pulmonary edema	3	Excl. of pleural effusion	7	Atelectasis	3
5	–	–	–	–	Enlarged heart	2	Pleural effusion	6	Healed rib fracture	3
**Arm ULDCT: Detection rate of ULDCT–findings and diagnoses stratified according to clinical relevance**										

Rank	Main diagnoses	27	Important incidental diagnoses	14	Strongly contributing findings	31	Moderately contributing findings	196	Clinically irrelevant findings^a^	278

1	Pneumonia	16	Dynamic of known lung cancer	2	Excl. of pneumothorax	9	Excl. of pneumonia	29	Coronary atherosclerosis	54
2	Pneumothorax	3	Aortic dilatation	2	Excl. of pneumonia	6	Excl. of pulm. edema	21	Aortic atherosclerosis	50
3	Pulmonary mass	2	Mult. solid pulm. nodules >8 mm	1	Excl. of pulm. edema	6	Pleural effusion	17	Calcified pulm. granuloma	20
4	Atelectasis	1	Pulmonary consolidation	1	Excl. of other referral diagnosis	3	Bronchial wall thickening	16	Atelectasis	18
5	Pleural effusion	1	Kidney lesion	1	Ground-glass opacities	2	Excl. of pneumothorax	13	Emphysema	14

To avoid an intra-reader bias, the determination of the detection rate was derived exclusively from the first imaging modality of the respective case.

Notably, findings and diagnoses were stratified with regard to their individual clinical relevance for every patient within the respective hospital visit.

a Clinically irrelevant for the respective hospital visit at the emergency department.

The observed advantages of ULDCT are more pronounced when it is used as an additional imaging modality after CXR. ULDCT added significantly more findings and diagnoses (n = 365) to prior CXR reports than vice versa (n = 32, *P* < 0.001), of which 354 ULDCT and 17 CXR findings and diagnoses were verified in the CompRS resulting in a significantly higher PPV for ULDCT of 97.0% CI [94.7, 98.3] vs. 53.1% CI [36.4, 69.1] for CXR (*P* < 0.001). ULDCT added 9.1% (CI [5.2, 15.5]; 11/121) of verified ‘main diagnoses’ to prior CXR reports. CXR, on the other hand, did not add a single verified ‘main diagnosis’ (0/134; *P* < 0.001) to prior ULDCT reports. ULDCT also contributed 50% (CI [30.7, 69.3]; 11/22) of verified ‘important incidental diagnoses’ vs. 0% (CI [0.0, 14.9]; 0/22) added by CXR (*P* < 0.001). Detailed results are shown in Table 2, Table 4 and Fig. 4.

**Table 4:** Detection rate of CXR and ULDCT as additional imaging modality (CXR after ULDCT in ‘Arm ULDCT’ and ULDCT after CXR in ‘Arm CXR’).

Arm ULDCT: Additional findings and diagnoses added by CXR to prior ULDCT reports										
Rank	Main diagnoses	0	Important incidental diagnoses	0	Strongly contributing findings	2	Moderately contributing findings	6	Clinically irrelevant findings^a^	9
1	–	–	–	–	Enlarged heart	1	Pulmonary edema	3	Aortic atherosclerosis	4
2	–	–	–	–	Pulmonary edema	1	Bronchial wall thickening	2	Atelectasis	1
3	–	–	–	–	–	–	Enlarged hilus	1	Healed rib fracture	1
4	–	–	–	–	–	–	–	–	Pleural thickening	1
5	–	–	–	–	–	–	–	–	Vertebral fracture	1
**Arm CXR: Additional findings and diagnoses added by ULDCT to prior CXR reports**										

Rank	Main diagnoses	11	Important incidental diagnoses	11	Strongly contributing findings	3	Moderately contributing findings	84	Clinically irrelevant findings^a^	245

1	Pneumonia	5	Enlarged thoracic lymph nodes	2	Pulmonary edema	2	Bronchial wall thickening	18	Coronary atherosclerosis	54
2	Bronchiolitis	2	Lymphadenopathy (malignant)	2	Patchy consolidations	1	Ground-glass opacities	8	Aortic atherosclerosis	22
3	Vertebral fracture	1	Solitary solid pulm. nodule >8 mm	2	–	–	Emphysema	7	Emphysema	16
4	Emphysema	1	Pulmonary trunk dilatation	1	–	–	Coronary atherosclerosis	6	Calcified pulm. granuloma	16
5	Bronchiectasis	1	Solitary solid pulm. nodule 6–8 mm	1	–	–	Excl. of bronchial wall thickening	6	Atelectasis	12

Notably, findings and diagnoses were stratified with regard to their individual clinical relevance for every patient within the respective hospital visit.

a Clinically irrelevant for the respective hospital visit at the emergency department.

Notably, ULDCT detected a higher proportion of clinically irrelevant findings for the respective hospital visit than CXR as both a primary modality and as an additional imaging modality with 78.5% (CI [74.0, 82.5]; 278/354) vs. 16.2% (CI [12.7, 20.3]; 58/359) and 68.2% (CI [63.3, 72.8]; 245/359) vs. 2.5% (CI [1.3, 4.7]; 9/354), both statistically significant (*P* < 0.001).

Although this trial assessed the detection rate of clinical relevance categories in a low-prevalence ED cohort, the incidence of pneumonia was sufficient to provide sensitivity, specificity, PPV and NPV as additional measures for this specific disease. As first imaging modality, ULDCT was superior to CXR with a sensitivity of 0.941 CI [0.730, 0.990] (16/17), specificity of 0.985 CI [0.946, 0.996] (128/130), PPV of 0.889 CI [0.672, 0.969] (16/18) and NPV of 0.992 CI [0.957, 0.999] (128/129) in comparison to CXR with a sensitivity of 0.625 CI [0.386, 0.815] (10/16), specificity of 0.939 CI [0.884, 0.969] (123/131), PPV of 0.556 CI [0.337, 0.754] (10/18) and NPV of 0.953 CI [0.902, 0.979] (123/129). Notably, the CompRS contained a total of 33 verified pneumonias, of which 31 were regarded as a main diagnosis for the individual patient and respective ED visit.

#### Exclusion performance of CXR and ULDCT

The referring diagnosis was excluded by CXR reports in 103 of the 147 patients in arm CXR, of which 91.3% (CI [84.2, 95.3]; 94/103) were correct exclusions while 8.7% (CI [4.7, 15.8]; 9/103) referring diagnoses were falsely ruled out. Among the 94 correct exclusions, 25 (26.6%) constituted a strongly and 68 (72.3%) a moderately contributing finding to the clinical main diagnosis, while one (1.1%) turned out to be clinically irrelevant.

ULDCT in arm ULDCT, on the other hand, recorded 112 exclusions with 97.3% (CI [92.4, 99.1]; 109/112) correct and only 2.7% (CI [0.9, 7.6]; 3/112) false. Although the absolute number of false exclusions was low for both modalities and the difference was not statistically significant (*P* = 0.053), the results indicate a higher reliability for correct exclusions by ULDCT. The clinical relevance of the correct exclusions was rated as a strongly contributing finding in 24 (22.0%) and as a moderately contributing finding in 84 (77.1%) patients. Only one (0.9%) exclusion was considered clinically irrelevant. Detailed results are shown in Table 2.

### Changes in patient management

If used as primary imaging modality, management was changed after CXR in 10.2% of patients (arm A; CI [6.3, 16.2]; 15/147) and after ULDCT in 32.0% (arm B; CI [25.0, 39.9]; 47/147) (*P* < 0.001). As an additional imaging modality, CXR has triggered management changes for 2.0% of patients (CI [0.7, 5.8]; 3/147), while additional ULDCT after prior CXR altered management in 28.6% of patients (CI [21.9, 36.3]; 42/147). Examples for management changes after additional ULDCT are provided in the Supplementary Materials.

### Radiation dose

The measured mean dose area product (DAP) for CXRs of the average patient in this trial was 3.7 dGy∗cm^2^ (±2.9), corresponding to a mean effective radiation dose of 0.05 mSv (±0.04). The mean radiation dose of a CXR in this trial was, therefore, approximately half the mean radiation dose of a standard CXR examination in the USA. Depending on mobility and clinical condition, patients were examined in one (n = 19) or two views (n = 275). The mean total dose length product (DLP) for the ULDCT scans, including the scan projection radiograph (scout view), was 12.7 mGy∗cm (±4.2). This corresponds to a mean effective dose of 0.22 mSv (±0.07), and therefore, approximately 4.5 times the mean effective dose of a clinical CXR at our institution or twice the radiation dose of a standard CXR in two views (mean dose levels). Detailed information is provided in the Supplements (Table E7, Figure E1).

### In-room time and reporting time

ULDCT required a longer mean in-room time (5.1 vs. 2.1 min) and mean reporting time (4.8 vs. 1.8 min) than CXR (Supplements Table E8).

### Follow-up examinations

As a primary imaging modality, four (2.7%) additional imaging examinations were recommended by CXR and 20 (13.6%) by ULDCT (Supplements Table E9).

## Discussion

In this prospective crossover trial of a low-prevalence non-traumatic emergency department patient cohort, we found that, on an intra-patient level, the detection rate for clinically relevant findings and diagnoses of ultra-low-dose CT (ULDCT) of the chest is superior to chest X-ray (CXR) at a radiation dose equal to approximately 4.5 clinical CXRs at our institution. As a primary imaging modality, ULDCT offered about twice the detection rate of CXR for main diagnoses and moderately contributing findings. Furthermore, ULDCT significantly increased the overall detection rate for main diagnoses when used as an additional modality after CXR, while CXR after ULDCT was unable to detect any further main diagnoses. This superiority is also reflected in the moderately contributing findings, important incidental diagnoses, rule outs and triggered management changes, for the use as primary as well as additional imaging modality.

Notably, ULDCT detected more than twice as many findings classified as clinically irrelevant for the respective hospital visit than did CXR, which might be considered a disadvantage of this imaging method. However, it is important to point out that these findings might still be relevant for further diagnostic workup and non-acute therapy of these patients (e.g., coronary atherosclerosis, calcifications of cardiac valves, pulmonary emphysema).

For our trial cohort, the most frequent ‘main diagnosis’ detected by both imaging methods was ‘pneumonia.’ Beyond a significantly higher detection rate for pneumonia itself, ULDCT contributed several additional intrapulmonary (e.g., bronchiolitis and intrapulmonary masses) as well as extrapulmonary ‘main diagnoses,’ while almost all ‘main diagnoses’ directly found by CXR were limited to pneumonia, confirming the limited value of CXR in non-traumatic ED patients.

To date, several studies have compared ULDCT and low-dose CT (LDCT) to standard-dose CT (SDCT), focusing on specific findings. These studies found good to excellent sensitivities and specificities for pulmonary nodules, pneumothorax, consolidations, ground-glass opacities, bronchiectasis, emphysema, honeycombing, and asbestos-related lung diseases.^17^^,^^23^, ^24^, ^25^^,^^28^^,^^30^, ^31^, ^32^, ^33^, ^34^, ^35^, ^36^ However, to our knowledge, only very few studies have directly compared ULDCT to CXR as an alternative primary imaging modality.

The assessment of the efficacy of diagnostic imaging has a long history in the radiologic community. In 1991, Fryback, Thornbury et al. published a hierarchical model as an organising structure for the appraisal of efficacy studies in diagnostic medical imaging.^37^ This scale categorises efficacy studies of imaging tests into the following six levels: Level 1 “technical efficacy,” level 2 “diagnostic accuracy efficacy,” level 3 “diagnostic thinking efficacy,” level 4 “therapeutic efficacy,” level 5 “patient outcome efficacy,” and level 6 “societal efficacy.” We emphasise that these levels should be used for categorisation of the respective study to provide comparability to existing literature and do not reflect the value of a study. Therefore, we provide the level of efficacy according to the Fryback–Thornbury efficacy scale for each study cited in this discussion.

Kroft et al.^38^ conducted a study (partially including Fryback–Thornbury Level 2, 3 and 4) with 200 patients referred by outpatient clinics or general practitioners, and, per study design, assessed the added value of ULDCT (mean effective dose [mED]: 0.07 mSv) after CXR. They found that the diagnostic confidence of the radiologist increased from 88% for the CXR to 98% after the additional ULDCT. Furthermore, the authors reported management changes caused by the additional information of ULDCT in 40 patients (20%). In contrast, our trial assessed the primary detection rate of both modalities, their additional value if used as second imaging modality, an analysis of exclusions of main referring diagnoses, and the clinical relevance and contribution of CXR and ULDCT to the final clinical diagnoses, while taking into account that not all of them are radiologically visualizable.

One of the most recent studies comparing ULDCT (mED: 0.05 mSv) and CXR was published by Taekker et al.^30^ in 2021 (Fryback–Thornbury Level 2). It prospectively included 91 emergency department patients with a clinical indication for a CXR in supine view (sCXR), who received a ULDCT and non-contrast SDCT of the chest in addition to the indicated sCXR. The authors retrospectively assessed four predefined chest conditions, whereas in our trial all patients with an indication for a CXR were eligible. Taekker et al. reported superior sensitivity for ULDCT compared to sCXR in the detection of pneumonia (90–100% vs. 50–80%) and pneumothorax (25–50% vs. 0%), while sensitivity was similar for pulmonary edema (0–56% vs. 0–78%) and pleural effusion (48–72% vs. 59–66%).

In contrast to these studies, a recent multicentre trial with 2418 non-traumatic ED patients by van den Berk et al.^39^ (OPTIMACT study group; Fryback–Thornbury Level 4 and 5) was designed as a non-inferiority trial with the focus on short-term patient outcomes after examination by either ULDCT or CXR. The authors found no inferiority of ULDCT, and only minimal differences in length of ED stay, hospital admissions, length of hospital stay, functional health at 28 days, as well as mortality rates between the two imaging modalities. Due to the wider availability and lower costs, they recommended the continued use of CXR as a primary modality in such an ED patient cohort. While both trials, van den Berk et al. and ours, assessed ULDCT and CXR in non-traumatic ED patients, they starkly differ regarding the trial design, including the outcome measures and time points of their assessment. In our trial, detection rates of clinical diagnoses, the clinical relevance, and exclusion performance were measured at the beginning of the diagnostic workflow, while van den Berk et al.^39^ measured the length of the hospital stay, functional health at 28 days, and mortality. The design of the latter trial, where patients would receive either CXR or ULDCT, prevented an intra-patient comparison of the modalities and may have resulted in a lower certainty for diagnoses. Furthermore, as described by the authors, the advantages of CXR with regard to time and costs may be limited by the significantly higher number of patients who required additional imaging examinations after CXR than after ULDCT. Therefore, the results cannot be directly compared. However, both trials shine light on the primary imaging modalities for non-traumatic ED patients from different angles and may contribute to further evidence-based discussion on this topic.

In contrast to the discussed literature, in our trial, assessed diagnoses were not limited to radiologically visible diagnoses, each finding and diagnosis was assigned to an individual clinical relevance category, and patients were eligible for participation regardless of indication for CXR, pre-existing conditions, or BMI. On the hierarchical Fryback–Thornbury scale^37^ we would categorise this trial as Level 3 and 4. Further strengths of our trial are that all patients were examined with both imaging modalities,^39^ CXRs were not limited to supine view or to the assessment of specific groups of diagnoses or findings.^30^

Regarding radiation protection, it is important to emphasize that this trial analysed ULDCT (only twice the radiation dose of a standard CXR) as an imaging alternative to CXR in non-traumatic emergency department patients, and both modalities assessed in this trial are at the lowest bound of radiation exposure in diagnostic medical imaging. At this level, radiation risks are so low that they are not measurable by epidemiological studies.^40^^,^^41^ By way of illustration, the mean radiation dose of 0.22 mSv for a ULDCT in this trial, 0.1 mSv for a standard CXR examination, and 0.05 mSv for a CXR in this trial, correspond to 26, 12, and six days of natural background radiation, respectively.^3^^,^^42^

This trial has several limitations. While an intra-reader bias was eliminated by using only the first imaging modality of the respective arm for the determination of the detection rate of CXR and ULDCT, the assignment of the clinical relevance categories might be prone to a confirmation bias. In order to reduce this potential bias, final clinical diagnoses were determined by experienced emergency physicians. Also, this trial focused on the clinical relevance of diagnoses and findings by imaging, and therefore, objective and subjective image quality were not evaluated. In addition, patient inclusion regardless of indication resulted in a large variety of diseases, a limited number of patients per disease, and a substantial proportion of patients without abnormal findings. Furthermore, this was a single-centre trial, but in-room time and reporting time are expected to vary among institutions due to different organizational frameworks and workflows in radiology departments. Since the main inclusion criteria was an indication for CXR only, the trial protocol did not include a standard-dose CT, which might have revealed additional findings. However, a comparison of ULDCT to SDCT was beyond the scope of this trial. Finally, the observed higher number of clinically irrelevant findings in reports of ULDCT compared to CXR may lead to unnecessary follow-up examinations.

Although this trial identifies ULDCT as clearly superior compared to CXR, a broader substitution of ULDCT for CXR may not yet be viable due to the increased reporting times, considering the already pressured personnel resources in radiology.^43^, ^44^, ^45^ Further studies are necessary to determine the rationale and economic aspects of such a substitution and, especially, to determine whether the observed benefits would justify the additional efforts.

In conclusion, ULDCT of the chest proved to be superior to CXR in a low-prevalence non-traumatic ED outpatient patient cohort. While ULDCT required a radiation dose of approximately 4.5 clinical CXRs at our institution or only two standard CXR examinations, the observed advantages with regard to the detection rate for ULDCT, including the doubling in the percentage of directly detected main diagnoses, support its use as one of the primary imaging modalities in the ED in the future. Further economic analyses will be necessary to determine the optimal spectrum of indications, rationale, and economic potential of a broader substitution of ULDCT for CXR as a primary imaging modality of the chest.

## Contributors

Christian Wassipaul – Conceptualization, Data curation, Formal Analysis, Funding acquisition, Investigation, Methodology, Project administration, Resources, Supervision, Validation, Visualization, Writing – original draft, Writing – review & editing.

Karin Janata-Schwatczek – Conceptualization, Data curation, Investigation, Methodology, Validation, Writing – review & editing.

Hans Domanovits – Conceptualization, Data curation, Investigation, Methodology, Validation, Writing – review & editing.

Dietmar Tamandl – Conceptualization, Data curation, Investigation, Methodology, Validation, Writing – review & editing.

Helmut Prosch – Conceptualization, Data curation, Investigation, Methodology, Validation, Writing – review & editing.

Martina Scharitzer – Data curation, Investigation, Writing – review & editing.

Stephan Polanec – Data curation, Investigation, Writing – review & editing.

Ruediger E. Schernthaner – Data curation, Investigation, Writing – review & editing.

Thomas Mang – Data curation, Investigation, Writing – review & editing.

Ulrika Asenbaum – Data curation, Investigation, Writing – review & editing.

Paul Apfaltrer – Conceptualization, Data curation, Investigation, Methodology, Validation, Writing – review & editing.

Filippo Cacioppo – Data curation, Investigation, Writing – review & editing.

Nikola Schuetz – Data curation, Investigation, Writing – review & editing.

Michael Weber – Conceptualization, Formal Analysis.

Peter Homolka – Methodology, Writing – review & editing.

Wolfgang Birkfellner – Methodology, Writing – review & editing.

Christian Herold – Resources, Supervision, Writing – review & editing.

Helmut Ringl – Conceptualization, Data curation, Formal Analysis, Funding acquisition, Investigation, Methodology, Project administration, Resources, Software, Supervision, Validation, Visualization, Writing – original draft, Writing – review & editing.

Christian Wassipaul and Helmut Ringl have accessed and verified all data.

## Data sharing statement

The anonymized participant data on which this study is based will be made available upon request by email to the corresponding author by qualified researchers after publication. No imaging data exceeding the images provided in this manuscript can be made available. Proposals will be approved by the authors on the basis of scientific merit and absence of competing interests after the signing of a data access agreement and confidentiality agreement.

## Declaration of interests

The Department of Biomedical Imaging and Image-guided Therapy of Medical University of Vienna has grants and contracts with more than 100 partners (organizational, academic, industry), all through official contracts with the Medical University of Vienna. Among these was funding from Siemens Healthineers (Erlangen, Germany) to employ two research assistants for one year for this study as well as grants independent of this study. CW was employed as research assistant by Medical University of Vienna for one year, enabled by funding from Siemens Healthineers and furthermore reports support for congress fees, travel and accommodation costs, unrelated to this study, by Medical University of Vienna. DT reports consulting fees from Roche and Siemens Healthineers, support for attending meetings and/or travel from Siemens Healthineers and participation on the DSM board, all unrelated to this study. HP reports honoraria as a speaker from AstraZeneca, BMS, Boehringer Ingelheim, Janssen, MSD, Novartis, Roche, Sanofi, Siemens Healthcare and Takeda as well as participation on the advisory board of AstraZeneca, Boehringer Ingelheim, Janssen, MSD and Sanofi, all unrelated to this study. MS reports support for congress fees, travel and accommodation costs, unrelated to this study, by Medical University of Vienna. RES reports honoraria as an educational speaker from Siemens Healthineers and a pending patent developed with Siemens Healthineers, all unrelated to this study. PA reports honoraria as a speaker from Siemens Healthineers, unrelated to this study. WB reports unpaid participation on the editorial board of Medical Physics and Zeitschrift fuer Medizinische Physik, both unrelated to this study. CH reports unpaid participation on the Photon Counting CT advisory board of Siemens Healthineers as well as stock ownership of Hologic until 2021, all unrelated to this study. HR was the PI of grants to the Medical University of Vienna from Siemens Healthineers until June 2018 and is still scientifically involved in several studies concerning these grants, but did not and does not receive remuneration nor is he part of the contracts; HR further reports honoraria as a clinical speaker from Siemens Healthineers until December 2019 and unpaid participation on the editorial board of European Radiology. The other authors declare no further competing interests.

## Appendix A Supplementary data

Supplementary Material

Chest Study Protocol

Figure E1
