# Supplementary material for: Ultra-low-dose CT vs. chest X-ray in non-traumatic emergency department patients – a prospective randomised crossover cohort trial
Source: eClinicalMedicine. 2023 Oct 17;65:102267. doi: 10.1016/j.eclinm.2023.102267 (PMC10590727; doi:10.1016/j.eclinm.2023.102267)
Supplement: Supplementary Material [file mmc1.pdf]

Supplementary material

Setting

This trial was conducted at the Department of Biomedical Imaging and Image-guided Therapy and the Department of Emergency Medicine (ED) at the Medical University of Vienna. Austria—a large tertiary medical center in central Europe with more than 1,600 beds, which manages more than 1·2 million outpatient cases annually in Vienna, a city of 1·9 million within an urban area of 2·9 million inhabitants.[1–4]

Compound reference standard

In a first step, an expert CT and chest radiologist with 23 years of experience (H.R.) assessed all prospective radiological reports for CXR and ULDCT from this trial, as well as all other available imaging and clinical data up to six months after the end of recruitment to come to a conclusive expert radiologist report for every patient. For seven cases, a second opinion was obtained from a chest radiology expert with 18 years of experience (H.P.) and a consensus was reached. Clinical diagnoses were alternately reviewed by two expert emergency physicians with more than 30 years of experience each (H.D., K.J.-S.) based on all available clinical data, including all imaging reports, to reach a conclusive expert diagnosis for each patient. In 21 ambiguous cases the respective other ED expert was consulted to reach a consensus.

In a second step, the expert radiologist (H.R.) and expert emergency physicians (H.D. and K.J.-S.) reached an agreement for all findings and diagnoses of every patient to define the final compound reference standard (CompRS) based on all available imaging and clinical data up to six months after the end of the recruitment period. Notably, the CompRS contains radiological findings as well as clinical diagnoses, including clinical diagnoses those that are undetectable by imaging methods.

In our study cohort, in total, 181 unambiguous different findings and diagnoses were defined as a base for comparison. For each of the 294 patients, all these possible findings and diagnoses were defined as true or false in the CompRS resulting in a database of 53214 entries.

The Compound Reference Standard (CompRS, all imaging findings and clinical diagnoses) contained a total of 1580 positive elements of these 53214 entries, corresponding to 1580 confirmed findings and diagnoses, resulting in a mean of 5·4 elements per patient, which showed no significant difference (P=0·746) between arm CXR (5·3

±2·7) and arm ULDCT (5·4 ±3·0). It is important to note, that these numbers simply show an equal distribution of findings in both trial arms and should not be mistaken for the detection rate of CXR and ULDCT.

Rationale for the application of clinical relevance categories

While the resultant set of findings or diagnoses allows for a bias-free dichotomous comparison between the reference standard and the assessed imaging modalities, an exclusive dichotomous approach hampers the inclusion of the extent or degree of a finding or diagnosis. If, e.g., extensive coronary calcifications are present in a report and the reference standard describes only mild calcifications, this approach rates it as a true imaging finding. The application of clinical relevance categories similar to those of Goldman et al. not only allows a clinical classification of imaging findings, but also solves this limitation of comparability because it will also rate this finding true, but, for most cases, clinically irrelevant.[5]

For this process, the expert radiologist and expert emergency physicians assessed all findings and diagnoses of the CompRS with regard to their individual clinical relevance for every patient within the respective hospital visit. The applied clinical relevance categories range from 'main diagnosis' that caused this ED visit via 'important incidental diagnosis' to 'strongly contributing', 'moderately contributing', and 'clinically irrelevant finding' for this ED visit. To avoid overemphasis of exclusions of main referring diagnoses, rule-outs were not classified as a 'main diagnosis', but, only as a 'strongly contributing finding' or a 'moderately contributing finding'. The following examples are provided to illustrate the application of the clinical relevance categories with regard to the respective ED visit for the individual patient.

A 69-year-old female patient, who presented with unspecific chest pressure received CXR and ULDCT. CXR did not reveal any sign of pneumonia and showed an elevated hemidiaphragm, as well as a unilaterally enlarged hilus. ULDCT also detected a markedly enlarged pulmonary trunk and extensive coronary artery calcifications. Additional laboratory testing confirmed elevated Troponin T and the patient was successfully treated with coronary angioplasty and stenting of the right coronary artery. In this case, the finding 'coronary artery calcifications' was rated as a 'moderately contributing' finding, since its detection by ULDCT contributed – in theory and in fact – to the correct diagnosis and treatment. However, for most other patients without correlating symptoms and/or laboratory test results, coronary artery calcifications, especially at lower extents, were rated as a 'clinically irrelevant finding' within the respective specific ED visit.

A 69-year-old male patient with known lung cancer who was undergoing radiotherapy presented with chest pain and newly elevated inflammatory markers without fever. CXR was indicated to search for the infection site. ULDCT showed the known pulmonary mass in the right lower lobe, regressive mediastinal lymph nodes (formerly PET-positive) and detected bilateral pulmonary patchy ground glass opacifications compatible with pneumonia, as well as a new right-sided adrenal mass in the partially depicted upper abdomen, which was suspicious for metastatic disease. CXR did not add any information to the prior ULDCT and was methodologically unable to detect the adrenal mass. The ground glass opacifications were rated as a 'strongly contributing finding' to the clinical 'main diagnosis' of pneumonia, while the suspicious adrenal mass was regarded as tumor progression, and therefore, as an 'important incidental diagnosis' for the respective ED visit. Subsequent PET-CT at a later hospital visit confirmed a metastasis of the right adrenal gland.

Power analysis

Assuming an accuracy of 80% for a CXR and 93% for ULDCT, calculations using NQuery Advanced (version 8.3.0.0) showed that 123 participants per arm (total of 246 participants) were required to reach a power of 85% in a two-sided test (alpha 5%) using a chi^2 test. Further assuming a dropout rate of 20%, the required sample size was calculated to be approximately 300.

Outcome measures and statistical analysis

In our study cohort, in total, 181 unambiguous different findings and diagnoses were defined as a base for comparison. For each of the 294 patients, all these possible findings and diagnoses were defined as true or false in the CompRS resulting in a database of 53214 entries. The Compound Reference Standard (CompRS, all imaging findings and clinical diagnoses) contained a total of 1580 positive elements of these 53214 entries.

The number of true and false positives were determined by dichotomous comparison of the prospective imaging report findings against the CompRS.

Sensitivity and specificity are, strictly speaking, reserved for tests of specific diseases rather than groups of diseases. Therefore, these terms should not be directly applied to the clinical relevance categories used in this trial. However, the main aim of this trial was to prospectively compare ULDCT and CXR for the detection rate of diagnoses according to their clinical relevance in a low-prevalence non-traumatic ED patient cohort.

While the term ‘sensitivity’ itself is not applicable to the clinical relevance categories used in our trial, the term ‘detection rate’ does not contain this limitation. Mathematically, both terms are calculated identically. The formula contains only true positives (TP) and false negatives (FN):

Sensitivity = (true positives) / (true positives + false negatives)

Specificity and NPV, on the other hand, require the ‘true negatives’ (TN) as a base. In total, in our trial, 181 different findings/diagnoses were observed. By design, our database contains a 0 or a 1 for all of these findings for each patient. However, if we would include the number of TN for all observed findings/diagnoses of all patients, the resulting specificity would be wrongfully skewed toward 1. On the other hand, reducing the TNs by using only a subset based on the clinical relevance categories is not appropriate due to the particular applicability of specificity to specific diseases.

Since the results would highly depend on the chosen subset of TN and might be misunderstood as a bias, we decided to use PPV instead. Therefore, to compare the performance of ULDCT and CXR, we used two measures, detection rate and PPV.

For both imaging modalities, as the primary and additional imaging modality, the PPV was calculated by comparing the findings of the respective imaging modality against the CompRS using the formula:

PPV = (number of true positives) / (number of true positives + number of false positives)

Changes in patient management – examples

The following examples are provided to illustrate management changes documented following a ULDCT report as additional imaging modality after prior CXR.

A 68-year-old female patient presented with unspecific chest pain and received a CXR examination to exclude pneumonia. CXR suspected a right-sided hilar mass with a diameter of 2 cm and recommended a standard chest CT for further workup. However, the additional ULDCT examination within this trial identified the suspected mass as a projection artefact, rendering the recommended standard chest CT irrelevant. In addition, it detected coronary and aortic sclerosis as well as an enlarged pulmonary trunk leading to the recommendation of cardiovascular and pneumological workup.

A 91-year-old female patient presented with an impaired general condition and a cough. She therefore received a CXR examination to confirm the suspected pneumonia with the differential diagnosis of an upper respiratory tract infection. CXR was unremarkable, apart from pronounced aortic sclerosis. The additional ULDCT report revealed centrilobular nodules and ground glass opacities in both posterior upper lobe segments as well as minor bronchial wall thickening and mucus plugging in the lingula. Therefore, ULDCT diagnosed bronchopneumonia potentially based on prior aspiration, initiating antibiotic treatment.

Device specifications and imaging acquisition protocols

ULDCT exams started with a scan projection radiograph (scout view) with tin filtration enabled and elevated arms at 50 mAs, 100 kVp, and a computed tomography dose index (CTDIvol) of 0·02 mGy. This scout view provided the attenuation information of the patient to the scanner and was required to calculate the appropriate tube current for the ULDCT scan. To keep the radiation dose of the ULDCT as low as possible, scout views were started at the suprasternal notch to avoid an upregulation of the dose modulation otherwise caused by the high attenuation of the shoulder girdle. The lower margin was the lateral costodiaphragmatic recess.

All CT images were acquired in helical scan mode and inspiratory breath-hold with the following parameters: tube voltage of 100 kVp; tin filtration enabled; tube current of 50 reference mAs; tube current modulation CAREDose (Siemens Healthineers, Erlangen, Germany) enabled in setting “weak”; collimation of 128 x 0.6 mm; gantry rotation time of 0·5 sec.; pitch of 1·2. The field of view was tailored to each patient’s requirements. These parameters were chosen to acquire the CT scans at a dose length product (DLP) of approximately 12·5 mGy*cm for a standard-sized patient, corresponding to a mean effective dose of 0·21 mSv.

CT images were reconstructed with a lung kernel (I50f) and a soft tissue kernel (I31f), both in coronal and transverse orientations at a section thickness (ST) of 3 and 1 mm and a reconstruction interval (RI) of 2 and 0·8 mm, respectively. For all kernels, the iterative reconstruction algorithm ADMIRE was used at iteration level 5 of 5 (Siemens Healthineers, Erlangen, Germany). For improved nodule detection, maximum intensity projection (MIP) reconstructions were computed at 12 mm ST and 3 mm RI. Standard lung and soft-tissue window settings were applied, but window level and width were freely adjustable for all readers. All images were transferred and stored to the local PACS system (IMPAX EE, Agfa HealthCare, Mortsel, Belgium) for prospective analysis and reporting.

Radiation dose

Radiation dose estimates were obtained from imaging and protocol data. For CXR, the dose area product (DAP, dGy*cm^2), and, for CT, the Volume Computed Tomography Dose Index (CTDIvol, mGy), as well as the Dose Length Product (DLP, mGy*cm), are provided.

To allow for intermodal comparison of radiation doses, the respective dose parameters were transformed to effective dose in mSv, which is widely used to compare radiation doses between imaging methods. The effective dose for ULDCTs for an average patient in this trial was calculated by multiplying DLP by the standard conversion factor for chest CTs of 0·017 mSv / (mGy*cm)[6], while the effective dose for CXRs was calculated by multiplying DAP by a conversion factor of 0·016 mSv/(dGy*cm^2) for pa-views, 0·013 for lateral views, and 0·020 for ap-views.[7] Since the concept of effective dose does not allow for the calculation of individual radiation risks, these were not calculated for the individual patient.

In-room time and reporting time

In this trial, the measured in-room time for both examinations was defined as the time a patient spent in the respective examination room.

Reporting time was recorded starting from the time at which the imaging data was first accessed until the electronic submission of the report. Reporting times were exclusively computed for the first of the two reports per patient, since reporting times for the second report were expected to be influenced by the prior report.

Recommended follow-up examinations

If used as the first imaging modality, a total of four additional imaging examinations were recommended by a CXR report (Table E9). Two of these were non-contrast-enhanced (non-CE), long-interval follow-up CTs of suspected pulmonary nodules and another two were short-interval non-CE CTs.

Reports on ULDCTs as a first imaging modality (Arm B) recommended a total of 20 additional imaging examinations. Seven of these were non-CE, long-term follow-up CTs, two contrast-enhanced (CE) long-term follow-up CTs, four short-term CE CTs, three immediate CT pulmonary angiographies, three mammograms / ultrasound examinations of breast lesions, and one sonography of a kidney lesion.

References – Online Data Supplement

1. Vienna General Hospital. Vienna General Hospital Annual Report 2019. Vienna General Hospital - Medical University Campus; 2020.

2. Mikulasek A, Fuchs R, Wisbauer A, editors. Demographisches Jahrbuch 2019. Vienna, Austria: Verlag Österreich GmbH; 2020.

3. European Commision, European Statistical Office. Eurostat - Data Explorer [Internet]. 2021 [cited 2021 May 15]. Available from: http://appsso.eurostat.ec.europa.eu/nui/submitViewTableAction.do

4. Müller M, editor. Taking Responsibility - Annual Report 2019. Vienna: Medical University of Vienna; 2020.

5. Goldman L, Sayson R, Robbins S, Cohn LH, Bettmann M, Weisberg M. The value of the autopsy in three medical eras. N Engl J Med. 1983 Apr 28;308(17):1000–1005.

6. Directorate-General for Research and Innovation (European Commission). European guidelines on quality criteria for computed tomography. Report EUR 16262. Brussels, Belgium: Publications Office of the EU; 2000 Feb p. 114.

7. Wall B, Haylock R, Jansen J, Hillier MC, Hart D, Shrimpton P. Radiation Risks from Medical X-ray Examinations as a Function of the Age and Sex of the Patient. Health Protection Agency United Kingdom; 2011 Oct.

Figure Legends – Online Data Supplement

Figure E1 – Thoracic Imaging, mean effective dose (mSv) for a typical standard-dose CT, low-dose CT, ultra-low-dose CT in the literature, ultra-low-dose CT in this trial, CXR in two views in the USA, as well as CXR in this trial.

Tables – Online Data Supplement

Table E1

Table E1 - Eligibility criteria

Inclusion criteria	Exclusion criteria
All non-traumatic ED-patients referred for a clinically indicated CXR	Severely critical condition
18-92 years of age	Recent prior imaging with the same referral diagnosis
Ability to provide informed consent	Pregnancy

Table E2

Table E2 – CXR acquisition parameters

	**Philips DigitalDiagnost system**	**Shimadzu MobileDaRt Evolution MX8c**
kVp for ap or pa / lat	90 (pa) / 125	96 (ap)
Automatic exposure control	On	n.a.
Added filtration	1 mm Al, 0·1 mm Cu	n.a.

Table E3

Table E3 – CT acquisition and reconstruction parameters.

**Siemens Somatom Drive**	
Tube voltage	100 kVp
Tin filtration	enabled
Tube current time product	50 ref. mAs
Tube current modulation CAREDose	enabled (weak)
Collimation	128 x 0·6 mm
Gantry rotation time	0·5 s
Pitch	1·2
Lung kernel	I50f, ADMIRE level 5 of 5
Soft tissue kernel	I31f, ADMIRE level 5 of 5
Slice thickness / reconstruction interval	1 / 0·8 mm (lung & soft tissue kernel)<br>3 / 2 mm (lung & soft tissue kernel)<br>12 / 3 mm (MIPs, lung kernel)

Table E4

Table E4 - Patients not eligible or excluded.

**Not eligible**	**No.**	**Excluded**	**No.**
Declined to take part	37	Severe dyspnoea in supine position	1
Inability to provide informed consent	16	Claustrophobia in CT gantry	1
Critical patient condition	2	Panic attack unrelated to CT scan	1
CXR was a follow-up	2		
Research associate was not informed	2		
Technical maintenance of CT scanner	1		
**Total**	**60**	**Total**	**3**

Table E5

Table E5 - BMI (grouped).

p=0·627			BMI (grouped)					Total
			<30	30 - ≤35	35 - ≤40	40 - ≤45	>45	
Arm	A	count	110	25	9	2	1	147
		%	74·8%	17·0%	6·1%	1·4%	0·7%	100·0%
	B	count	113	18	14	1	1	147
		%	76·9%	12·2%	9·5%	0·7%	0·7%	100·0%
Total		count	223	43	23	3	2	294
		%	75·9%	14·6%	7·8%	1·0%	0·7%	100·0%

Table E6

Table E6 – Compound Reference Standard (CompRS) for both arms. The ten most frequent findings and diagnoses in the CompRS as well as detected by CXR and ULDCT as a first or second imaging modality (non-exhaustive list). Notably, this CompRS contained radiological findings, as well as clinical diagnoses, including those undetectable by imaging methods.

CompRS for both arms - findings and diagnoses stratified according to clinical relevance, including radiologically undetectable diagnoses

Rank	Main diagnoses	314		Important incidental diagnoses	44		Strongly contributing findings	67		Moderately contributing findings	442		Clinically irrelevant findings *	713	
1	No main diagnosis found	59	18·8%	Coronary artery disease	4	9·1%	Excl. of pneumothorax	18	26·9%	Bronchial wall thickening	62	14·0%	Aortic atherosclerosis	119	16·7%
2	Musculoskel. thoracic pain	36	11·5%	Bronchitis	3	6·8%	Excl. of pneumonia	16	23·9%	Excl. of pneumonia	54	12·2%	Coronary atherosclerosis	118	16·5%
3	Pneumonia	31	9·9%	Lymphadenopathy (malignant)	3	6·8%	Excl. of pulm. edema	9	13·4%	Excl. of pulm. edema	38	8·6%	Atelectasis	42	5·9%
4	Cardiac decompensation	23	7·3%	COPD	2	4·5%	Pulmonary edema	7	10·4%	Pleural effusion	29	6·6%	Calcified pulm. granuloma	40	5·6%
5	Bronchitis	15	4·8%	Dynamic of known lung cancer	2	4·5%	Excl. of other referral diagn.	5	7·5%	Excl. of pneumothorax	25	5·7%	Pulmonary emphysema	34	4·8%
6	COPD exacerbation	9	2·9%	Aortic dilatation	2	4·5%	Enlarged heart	3	4·5%	Ground-glass opacities	19	4·3%	Linear pulm. opacities	31	4·3%
7	NSTEMI	7	2·2%	Pulmonary trunk dilatation	2	4·5%	Ground-glass opacities	2	3·0%	Pulmonary emphysema	18	4·1%	Bronchial wall thickening	28	3·9%
8	Fever of unknown origin	7	2·2%	Enlarged thoracic lymph nodes	2	4·5%	Bronchiolitis	2	3·0%	Excl. of bronchial wall thickening	16	3·6%	Solitary solid pulm. nodule <6mm	25	3·5%
9	Atrial fibrillation	6	1·9%	Solitary solid pulm. nodule >8mm	2	4·5%	Bronchial stenosis	1	1·5%	Pulmonary consolidation	14	3·2%	Calcification of cardiac valve	24	3·4%
10	Upper resp. tract infection	6	1·9%	Breast lesion	2	4·5%	Pulmonary emphysema	1	1·5%	Coronary atherosclerosis	13	2·9%	Mult. solid pulm. nodules <6mm	20	2·8%

*Clinically irrelevant for the respective hospital visit at the emergency department.

Table E7

Table E7 - Radiation dose of CXR and ULDCT in both study arms.

	Total		*P*
	**Arm A (CXR – ULDCT)**	**Arm B (ULDCT – CXR)**	
CXR<br>Dose Area Product (DAP, dGy*cm²; mean ± SD [range])	3·7 ± 2·9 (0·3 – 24·1)		
	4·0 ± 3·3 (0·3 – 24·1)	3·3 ± 2·4 (0·3 – 14·2)	0·089
CXR<br>Effective Dose (ED, mSv; mean ±SD)	0·050 ± 0·039		
	0·055 ± 0·044	0·046 ± 0·032	0·059
ULDCT<br>Dose Length Product (DLP, mGy*cm; mean ± SD [range])	12·7 ± 4·2 (5·2 – 34·3)		
	13·2 ± 4·5 (7·2 – 34·3)	12·2 ± 3·7 (5·2 – 26·9)	0·067
ULDCT<br>Effective Dose (ED, mSv; mean ± SD)	0·22 ± 0·07		
	0·22 ± 0·08	0·21 ± 0·06	0·067
ULDCT<br>Volume CT Dose Index (CTDIvol, mGy; mean ± SD [range])	0·37 ± 0·13 (0·15 - 0·95)		
	0·38 ± 0·13 (0·18 - 0·93)	0·36 ± 0·12 (0·15 - 0·95)	0·105
ULDCT<br>Size Specific Dose Estimate (SSDE, mGy; mean ± SD [range])	0·43 ± 0·10 (0·20 - 0·92)		
	0·44 ± 0·11 (0·20 - 0·92)	0·42 ± 0·09 (0·24 - 0·89)	0·111

Table E8

Table E8 - In-room time and reporting time of CXR and ULDCT in both study arms.

	**Total**		***P***
	**Arm A (CXR – ULDCT)**	**Arm B (ULDCT – CXR)**	
In-room time CXR (min:sec; mean ± SD [range])	02:03 ± 01:01 (00:44 – 06:26)		
	02:05 ± 01:01 (00:44 – 06:26)	02:01 ± 01:00 (00:45 – 05:12)	0·510
In-room time ULDCT (min:sec; mean ± SD [range])	05:08 ± 01:47 (02:08 – 12:45)		
	04:58 ± 01:36 (02:28 – 09:48)	05:16 ± 01:57 (02:08 – 12:45)	0·263
Reporting time CXR (min:sec; mean ± SD [range])	01:39 ± 01:03 (00:14 – 10:03)		
	01:45 ± 00:57 (00:14 – 05:58)	01:33 ± 01:08 (00:15 – 10:03)	0·010
Reporting time ULDCT (min:sec; mean ± SD [range])	04:46 ± 02:07 (00:20 – 14:58)		
	04:36 ± 02:06 (00:56 – 14:58)	04:57 ± 02:08 (00:20 – 10:52)	0·128

Table E9

Table E9 - Recommended follow-up examinations by CXR and ULDCT as primary imaging modalities.

	**CXR in Arm A**	**ULDCT in Arm B**
CT follow-up. non-CE	2	7
CT short-term. non-CE	2	0
CT follow-up. CE	0	2
CT short-term. CE	0	4
CTPA	0	3
Other imaging examinations	0	4
**Total**	**4**	**20**

Figures – Supplementary material

Figure E1

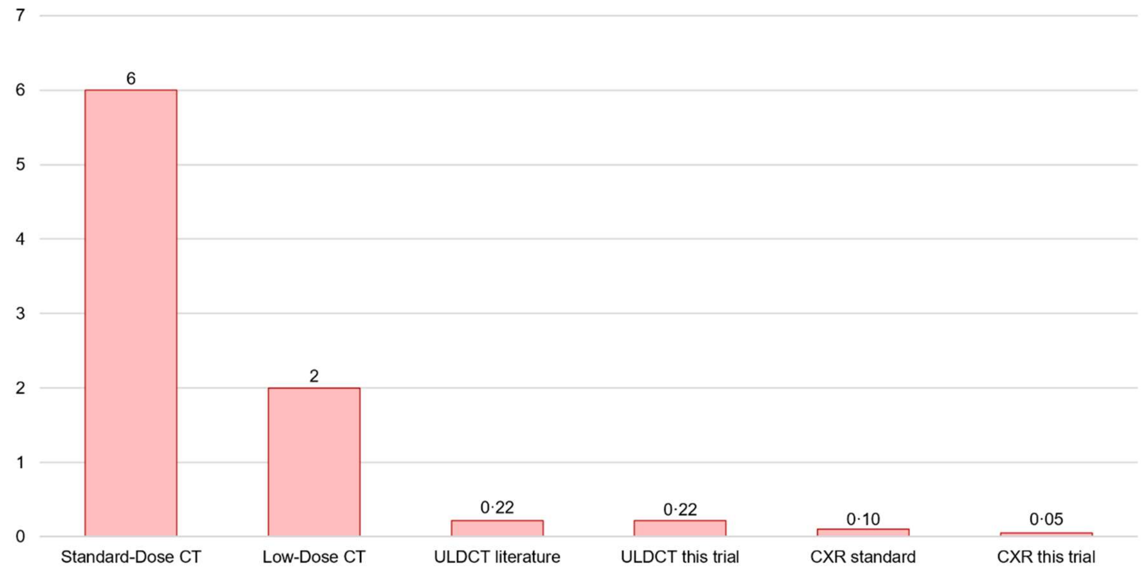

Figure E1 – Thoracic Imaging – mean effective dose (mSv). Mean effective dose of a typical standard-dose CT, low-dose CT, ultra-low-dose CT in the literature, ultra-low-dose CT in this trial, CXR in two views in the USA, as well as CXR in this trial.
