# Supplementary material for: Ultra-low-dose CT vs. chest X-ray in non-traumatic emergency department patients – a prospective randomised crossover cohort trial
Source: eClinicalMedicine. 2023 Oct 17;65:102267. doi: 10.1016/j.eclinm.2023.102267 (PMC10590727; doi:10.1016/j.eclinm.2023.102267)
Supplement: Chest Study Protocol [file mmc2.pdf]

Accuracy of Ultra-Low-Dose CT (ULDCT) of the Chest Compared to Plain Film in an Unfiltered Emergency Patient Cohort

Study protocol and statistical analysis plan

English Version

Version 1.3

24th of April 2019

Table of contents

1 AUTHORS 3

2 PARTICIPANTS 3

3 PROJECT TITLE 4

4 BACKGROUND AND SUMMARY 5

5 STUDY DESIGN 7

6 HYPOTHESES AND OUTCOME MEASURES 10

7 INCLUSION CRITERIA 11

8 EXCLUSION CRITERIA 12

9 ETHICAL CONSIDERATIONS 12

10 RECRUITMENT 13

11 STUDY DURATION 13

12 RADIATION DOSE 13

13 RADIOLOGICAL ANALYSIS 14

14 STATISTICAL ANALYSIS PLAN 15

15 COMPENSATION 15

16 STUDY SUSPENSION 16

17 DATA MANAGEMENT AND DATA PROTECTION 16

18 FUNDING 16

ULDCT project

1 Authors

- Univ.Prof. Univ.-Doz. Dr.med.univ. Helmut Ringl, MBA
- Mag.rer.soc.oec. Dr.med.univ. Christian Wassipaul
- Mag. Dr. Michael Weber
- ao.Univ.-Prof. Dipl.-Ing. Dr. Peter Homolka
- Ass.-Prof. Priv.-Doz. Dr.med.univ. Paul Apfaltrer, MBA
- Assoc.Prof. Priv.-Doz. Dr.med.univ. Dietmar Tamandl
- Dr.med.univ. Mathias Lazar
- Assoc.Prof. Priv.-Doz. Dr.med.univ. Helmut Prosch
- Assoc.Prof. Priv.-Doz. Dr.med.univ. Thomas Mang
- Assoc.Prof. Priv.-Doz. Dr.med.univ. Rüdiger Schernthaner
- Dr.med.univ. Ulrika Asenbaum
- o.Univ.-Prof. Dr.med.univ. Christian Herold
- ao.Univ.-Prof. Dr.med.univ. Hans Domanovits
- Ass.-Prof. Dr.med.univ. Karin Janata-Schwatczek
- Dr.med.univ. Sebastian Schnaubelt
- Dr.med.univ. Filippo Cacioppo

2 Participants

Intended number of participants	250
Limiting age, minimum	18 years
Limiting age, maximum	92 years
Ability to provide informed consent	Yes
Male participants	Yes

Female participants	Yes
Duration of participation in the trial for each participant	Approximately two hours for each patient from inclusion in the study until the therapeutic consequences are established.
Active phase	Six months
Follow-ups	Within the trial no follow-up is intended. However, if clinically indicated follow-ups are performed, this data may be used in the analysis phase of the study for final diagnosis.
Estimated duration of the trial	One year<br>(six months of acquisition, six months of evaluation)

3 Project title

Brief title

Accuracy of **U**ltra-Low-Dose-CT of the Chest Compared to **P**lain Film in an Unfiltered Emergency Department Patient Cohort

Acronym

UP-Chest

Official title

Accuracy of Ultra-low-dose-CT (ULDCT) of the Chest Compared to Plain Film in an Unfiltered Emergency Department Patient Cohort

4 Background and summary

For nearly a century, chest X-ray (plain film, projectional radiography) has been the established primary imaging modality for patients with acute chest pain, suspected pneumonia, and / or acute dyspnoea in the diagnostic pathway of emergency departments, although the sensitivity and specificity of this X-ray technique are moderate. (Long et al. 2017; Andronikou et al. 2017; Chalmers 2016; Martindale et al. 2016; Cardinale et al. 2014; Chawla et al. 2016)

The widespread availability and use of chest X-ray is due to the low acquisition and operating costs for projectional radiography equipment, the short examination time, and the very low radiation exposure. In addition, projectional radiography of the chest often serves as a guide for further, more sensitive, diagnostical procedures. However, these advantages are partially offset by the disadvantages inherent to projectional methods: anatomical structures may superpose or mask pathological structures. As a result, some areas of the lung may be obscured, and assessment may therefore be limited.

Whereas computed tomography was reserved for certain clinical questions over the last few decades, and, in most cases, served as a second imaging approach after plain film radiography, it has increasingly evolved as a primary imaging modality for several indications (e.g., suspicion of pulmonary embolism, highly suspected aortic dissection). This rise of computed tomography was due not only to its significant advantage of no superposition, but also partly driven by the marked reduction in radiation dose needed without sacrificing image quality. This was driven by the development of new detectors, modulation of tube current and voltage, as well as iterative reconstruction techniques. As a result, recent computed tomography scanners currently offer not only a more precise visualization of differences in tissue-attenuation and the significant advantage of the absence of artefacts due to superposition, but also allow for imaging with a considerably reduced radiation dosage compared to older scanners. (Zinsser et al. 2018; Anon 2014; Brenner and Hall 2007; Berrington de González et al. 2009) (O'Hora and Foley 2018; Moser et al. 2017; Kubo et al. 2014; Kubo et al. 2017) Therefore, computed tomography may now be utilized as screening method in specific indications that carry an increased risk of certain pathologies (e.g., in long-time smokers) (Horeweg et al. 2014; Walter et al. 2016; Yousaf-Khan,

van der Aalst, de Jong, Heuvelmans, Scholten, Lammers, et al. 2017; Yousaf-Khan, van der Aalst, de Jong, Heuvelmans, Scholten, Walter, et al. 2017; National Lung Screening Trial Research Team et al. 2011; van der Aalst et al. 2016; Ruchalski and Brown 2016; Fintelmann et al. 2017). The introduction of the latest generation of computed tomography devices about three years ago allowed for an even further reduction in dose by filtering out low-energy photons using a tin filter, which offers the possibility of a reduction in radiation dose by another 50 % or more for established CT indications. For specific indications (e.g., lung nodules in follow-up), the radiation dose may even be reduced to a tenth or one-hundredth of a standard-dose CT (SDCT). (Braun et al. 2015; Suntharalingam et al. 2018; Haubenreisser et al. 2015)

The current reference dose-length-product (DLP) in Germany for thoracic standard-dose CT (SDCT) is ~350 mGycm (effective dose ~6 mSv) and, for thoracic low-dose-CT (LDCT / HR-CT), ~100 mGycm (effective dose ~1.7 mSv) (Schegerer 2016). However, the latest devices (third-generation dual-energy CT) provide the opportunity to considerably reduce the reference dose of thoracic low-dose CT. In the current literature, these scans are referred to as Ultra-Low-Dose-CT (ULDCT) and are usually associated with a radiation dosage of 0.14 to 0.5 mSv. For this dose range, no standardized reference values have been published as yet. (Macri et al. 2016; Messerli, Ottilinger, et al. 2017; Messerli, Giannopoulos, et al. 2017; Messerli, Hechelhammer, et al. 2017; Vardhanabhuti et al. 2017; Martini et al. 2016; Rob et al. 2017; Moore et al. 2015; Braun et al. 2015)

The limiting factors of ULDCT are quantum noise, loss of spatial resolution, and other image artefacts (Kim et al. 2015). Therefore, careful selection of appropriate CT protocols and dosage is mandatory in order to achieve sufficient image quality to answer the respective diagnostic question.

Several papers have been published on the subject of ULDCT, which are dedicated to the comparison of ULDCT with LDCT and/or SDCT. These papers conclude that this technology may be used with sufficient sensitivity and specificity for indications such as dyspnea, emphysema, or lung nodules. (Macri et al. 2016; Messerli, Ottilinger, et al. 2017; Messerli, Giannopoulos, et al. 2017; Messerli, Hechelhammer, et al. 2017; Vardhanabhuti et al. 2017;

Martini et al. 2016; Rob et al. 2017; Moore et al. 2015) Due to the potential to reduce the radiation dose to less than 1/30 of a standard-dose CT while still providing acceptable image quality with the latest generation of devices, ULDCT of the chest is emerging as an interesting alternative to conventional chest X-ray.
To the best of the authors' knowledge, there are currently no studies comparing and evaluating ultra-low-dose-CT as a primary imaging alternative to chest X-ray in emergency department patients.

This study aims to compare ULDCT and plain film of the chest with regard to their accuracy in an unfiltered patient cohort of an emergency department. For this purpose, our ULDCT protocol will use the lowest possible dose at which image quality is diagnostically sufficient (approximately 0.2 mSv effective dose). This corresponds to less than 1/30 of the radiation dose of a standard-dose CT of the chest and to only about 2.5 times the dose of a chest X-ray in two views. This dose is equal to less than a month of natural background radiation in Austria and less than the radiation exposure on an intercontinental flight. (Bundesministerium für Arbeit, Soziales, Gesundheit und Konsumentenschutz 2018b; Bundesministerium für Arbeit, Soziales, Gesundheit und Konsumentenschutz 2018a; Ditto et al. 2013)

In addition to the accuracy of ULDCT of the chest compared to plain film of the chest, this trial also aims to analyze the clinical relevance of both methods by assessing the respective impact on final diagnosis, as well as possible changes in therapy.

5 Study Design

Study Type	Interventional
Primary Purpose	Diagnostic
Number of Arms	2
Masking (prospective)	None (open label)
Allocation	Randomized
Enrolment	250

Arms	Assigned Interventions
Reporting-order: Plain Film - ULDCT<br><br>The plain film of half the participants (randomized) will be submitted for reporting by a radiologist as a first imaging method. After finishing this report, the same radiologist will assess the ULDCT of this participant. In this second report, the findings of both examinations will be summarized, and a second report will be filed.<br><br>Emergency physicians will first receive the report for the plain film of the chest and will be asked for the diagnosis and its probability. Next, the report for ULDCT will be presented to them. Again, diagnosis and probabilities will be documented.	Diagnostic Test: ULDCT<br><br>Ultra-Low-Dose-CT (ULDCT) of the chest using tin filters with third-generation dual-energy CT devices. The projected dose used will be approximately 0.2 mSv per ULDCT of the chest.
Reporting-order: ULDCT - Plain Film<br><br>For half the participants (randomized) radiologists will first receive the data from ULDCT of the chest and write a report. Subsequently, they will receive the data from the plain film of the chest and may expand their report (explicitly separated).<br><br>Emergency physicians will first receive the report for the ULDCT of the chest and will be asked for probabilities of the nine most frequent diagnoses in chest-imaging plus "other". Next, they will be presented with the report for the plain film and will again be asked to give an estimation of the probabilities for the same diagnoses as before.	Diagnostic Test: ULDCT<br><br>Ultra-Low-Dose-CT (ULDCT) of the chest using tin filters with third-generation dual-energy CT devices. The projected dose used will be approximately 0.2 mSv per ULDCT of the chest.

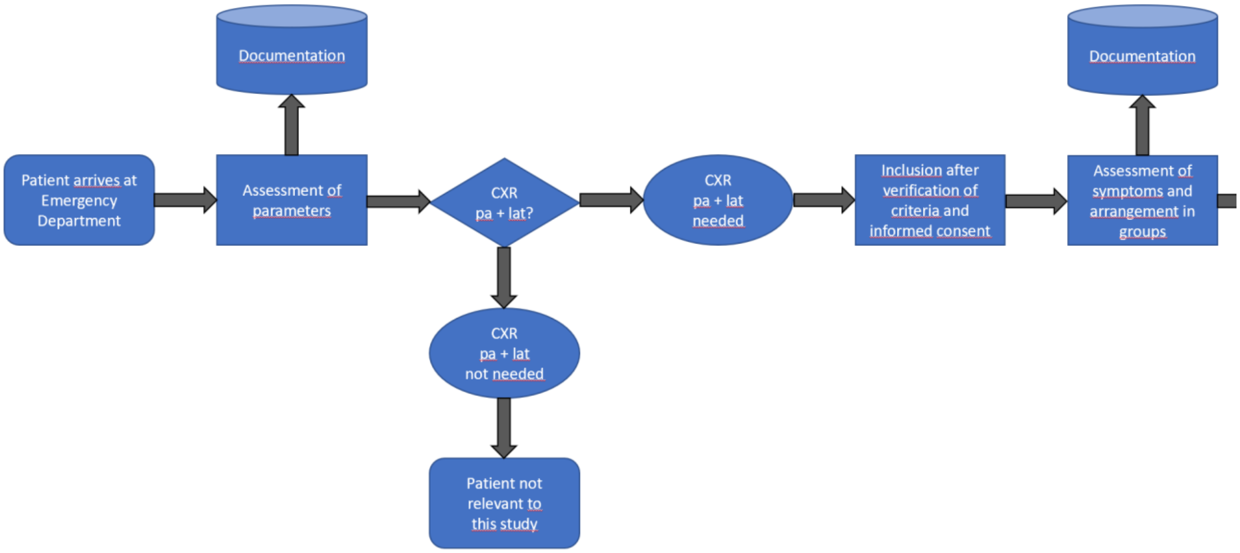
Documentation
Documentation
Patient arrives at Emergency Department
Assessment of parameters
CXR pa + lat?
CXR pa + lat needed
Inclusion after verification of criteria and informed consent
Assessment of symptoms and arrangement in groups
CXR pa + lat not needed
Patient not relevant to this study

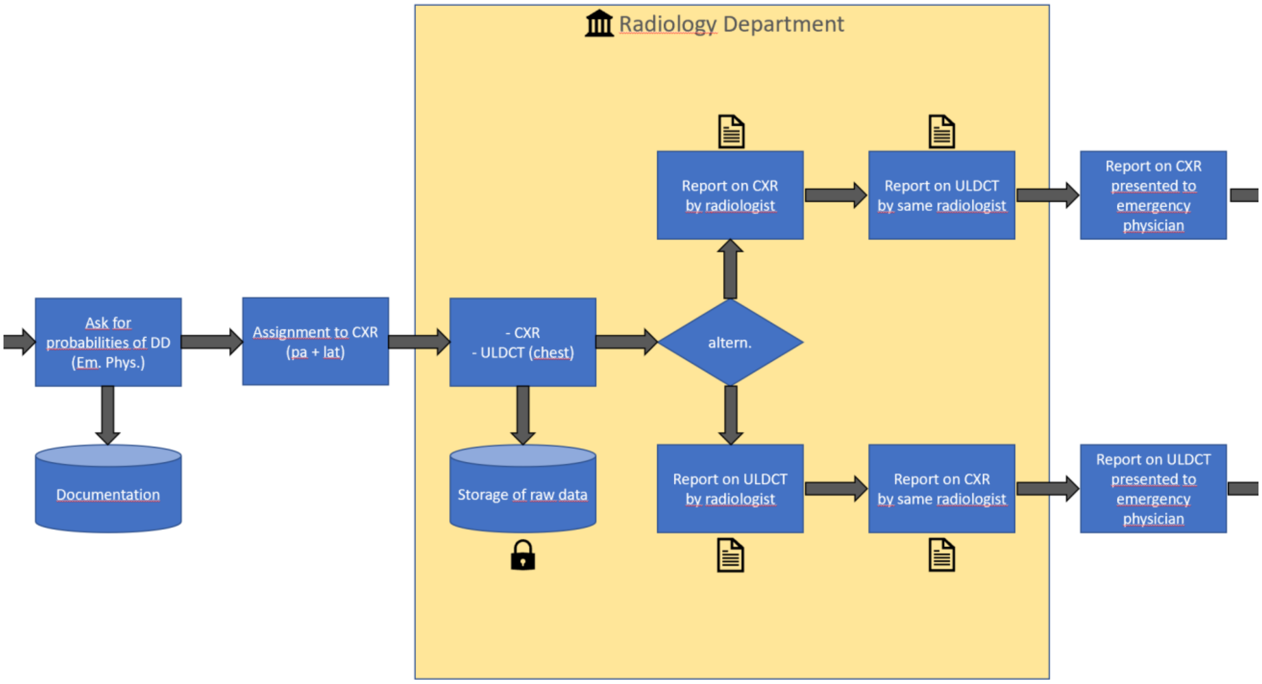
Radiology Department
Report on CXR by radiologist
Report on ULDCT by same radiologist
Report on CXR presented to emergency physician
Ask for probabilities of DD (Em. Phys.)
Assignment to CXR (pa + lat)
- CXR
- ULDCT (chest)
altern.
Documentation
Storage of raw data
Report on ULDCT by radiologist
Report on CXR by same radiologist
Report on ULDCT presented to emergency physician

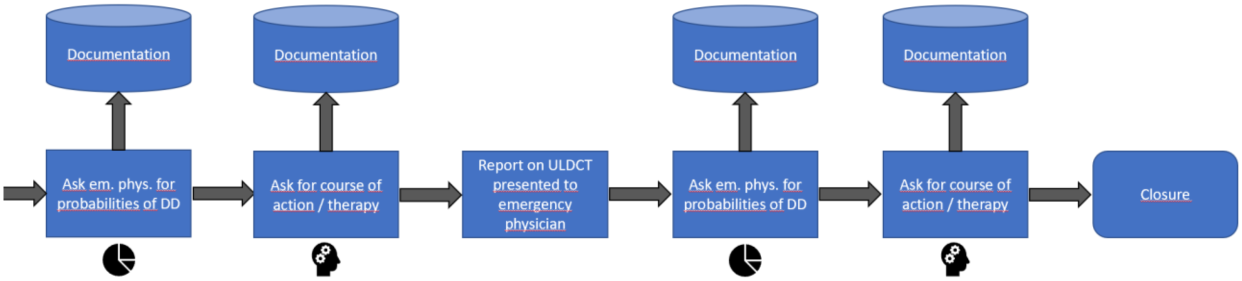

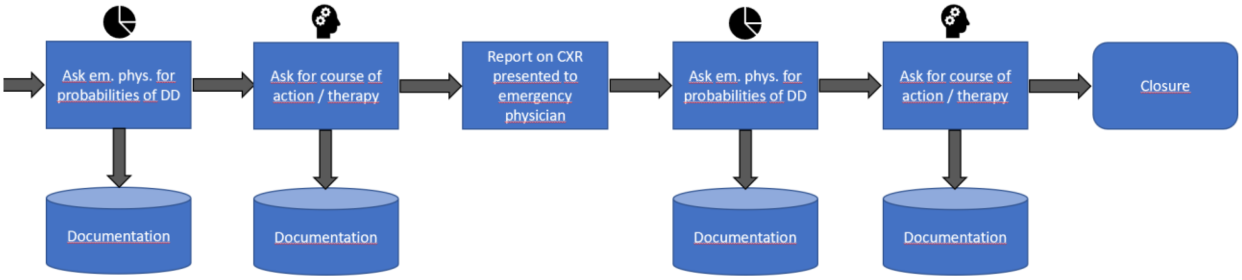

6 Hypotheses and outcome measures

Null hypothesis:

- There is no difference in accuracy between ULDCT of the thorax and plain film of the thorax regarding the primary diagnosis.

Alternative hypothesis:

- ULDCT of the thorax offers higher accuracy than plain film of the thorax regarding the primary diagnosis.

Primary Outcome Measure:

Accuracy of ultra-low-dose-CT of the chest and plain film of the chest

Description:

Initial radiologic diagnostic accuracy of both methods will be assessed by analyzing the number of reports that are changed after the images of the second modality become available to the radiologist in Arm 1 compared to Arm 2.

In a final approach, the diagnostic accuracy will be analyzed by retrospectively comparing all reports with the gold standard, which will be built from all available patient data at the end of the study, including all follow-up imaging studies and laboratory tests.

Secondary Outcome Measures:

- Sensitivity and specificity in ULDCT and plain film
- Frequency of change in radiological diagnosis
- Frequency of change in emergency physician's diagnosis
- Frequency of change in (planned) therapeutic course of action by emergency physician
- Frequency of accidental diagnosis in ULDCT of the chest and plain film of the chest
- Frequency of additional diagnostic imaging needed
- Frequency of unclear reports in ULDCT and plain film
- Diagnostic confidence in ULDCT and plain film by radiologist
- Diagnostic confidence in ULDCT and plain film by emergency physician

7 Inclusion criteria

Sex	All
Gender-based	No
Age limits	Minimum: 18 years<br>Maximum: 92 years
Accept healthy volunteers	No
Participants	All patients who are assigned to a clinically indicated chest X-ray by the emergency department of Vienna General Hospital
Consent	Ability to provide informed consent<br>Informed consent after detailed patient briefing

8 Exclusion criteria

Clinical status	A critical clinical condition that does not allow the examination with both modalities (ULDCT of the chest, chest X-ray)
Assignment	Assigned to chest X-ray as follow-up
Pregnancy	Women with positive ß-HCG-test

9 Ethical considerations

- The mean effective dose of a chest X-ray examination in two views (pa, lat) is 0.08 mSv (Wachabauer and Röthlin 2017).
- The targeted mean effective radiation dose for one thoracic ultra-low-dose-CT (ULDCT) is approx. 0.2 mSv. This corresponds to approximately one month of natural background radiation in Austria. Compared to standard-dose CT examinations, ULDCT of the thorax causes about one-twentieth to one-fortieth of effective radiation dosage. Therefore, its dosage is much closer to a chest X-ray in two views than to a standard-dose CT, or even a low-dose CT examination.
- Even the cumulative dose of both examinations comes with a negligible radiation dose. Thus, no negative effects are to be expected.
- A delay in diagnosis can be ruled out since each examination is promptly evaluated – before the examination for the second imaging modality is performed.
- Since no contrast medium is applied, adverse effects or events – in this context – can be ruled out.

10 Recruitment

All patients who are assigned to receive a clinically indicated chest X-ray by the emergency department of Vienna General Hospital – regardless of indication – are offered participation in this trial. Thus, only clinically indicated cases may be included.
Patients who meet the eligibility criteria and provide informed consent become participants in this trial and receive an ultra-low-dose-CT examination of the thorax in addition to a chest X-ray.

11 Study duration

Based on a preliminary analysis of frequency, the anticipated total study duration, including analysis of all parameters, is one year.

12 Radiation dose

- Chest X-ray: The reference dose for a chest X-ray in two views is approx. 0.08 mSv (effective dosage) (Wachabauer and Röthlin 2017).
- Thorax-ULDCT: based on recent publications regarding imaging in emphysema and dyspnea, as well as on measurements with a thorax-CT test-phantom performed with the specific CT devices used for the study (Siemens Somatom Drive and Siemens Somatom Force), an effective dose of approx. 0.2 mSv (corresponding to DLP 12.5) appears to be optimal for ULDCT (lowest dose possible with sufficient image quality). This dose is below the effective dose of one month of natural background radiation, which is referenced as 0.23 mSv per month in Austria (2.8 mSv per year). (Bundesministerium für Arbeit, Soziales, Gesundheit und Konsumentenschutz 2018a; Bundesministerium für Arbeit, Soziales, Gesundheit und Konsumentenschutz 2018b; Ditto et al. 2013)

The thorax-CT test-phantom used is model RS-111 by "Radiology Support Devices", Long Beach, California, USA.

13 Radiological analysis

Rationale behind the study design:

Prospective:
For ethical, practical, and legal reasons, the emergency physician always needs an immediate consensus report on both methods written by a single specialist in radiology in order to make a final treatment decision. This would not be possible without a significant delay, if two radiologists had to create two separate reports. The frequency with which diagnoses and therapies by radiologists and emergency physicians differ between Arm 1 and Arm 2 will be measured.

Retrospective:
This will involve a multi-reader analysis by four radiologists in training and four specialists. All eight physicians will independently read and report all examinations by means of structured reporting. The reading time is not specified. Two groups with all 300 patients each will be created: In the 1st group, the chest X-ray examinations of Pts. 1-150 and the Ultra-Low-Dose-CT examinations of Pts. 150-300 will be available. In the 2nd group, this will be reversed. All patients will be fully anonymized and randomized. Two radiologists in training and two specialists will first read and report on group 1, the other four doctors will first read and report on group 2. Between the evaluations of group 1 and group 2 there will be at least one month to prevent a possible recognition of a case.
The gold standard will be built from all available patient data at the end of the study, including the results of all imaging procedures, follow-up examinations, physical medical examinations, laboratory and histopathological tests and further diagnostic procedures (concerns all available data, regardless of whether the patient is admitted as an inpatient).
The structured findings are then compared with the gold standard to determine the accuracy, sensitivity and specificity of both procedures.

Documentation:

- Waiting time for the ULDCT will be logged.
- Waiting time for the X-ray will be logged.

14 Statistical analysis plan

Power and sample size:

A total of 250 participants is targeted.

This is based on the following assumptions:

- Accuracy of a chest X-ray is 80%
- Accuracy of a ULDCT of the chest is 93%
- Dropout rate is 20% (one in five potential participants)

According to calculations using NQuery Advanced (version 8.3.0.0), 123 participants per arm (total of approx. 250 participants) will be required to reach a power of 85% in a one-sided test (alpha 2.5%). Due to the assumption of a dropout rate of 20% (approx. one in five potential participants), a sample size of 300 is required.

Cross tabulation and chi^2-tests will be used to compare percentages (e.g., accuracy, sensitivity, …). Diagnostic confidence will be compared by applying a Mann-Whitney U test.

15 Compensation

None.

Participants will not receive any compensation.

16 Study suspension

Since no negative effects on patients are expected, the trial will be suspended or stopped only if it becomes obvious that ultra-low-dose-CT examinations do not offer any benefit.

17 Data management and data protection

Only patients with clinical indications for chest X-rays will be included. During the active diagnostic phase, these patients will be diagnosed and treated in the usual clinical setting and documented using the RIS (Radiology Information System). Thus, in this part of the study, open data will be used.

The evaluation of prospective and retrospective data will be subject to complete anonymization and randomization and will not be un-blinded at any time thereafter. Anonymization is performed by the vb.net - RND function, local software, and complete deletion of all personal, public, and private DICOM tags in the header.

The data will remain at the Medical University of Vienna (MUW) and the Vienna General Hospital (AKH Vienna). Backup copies, which will remain in-house, will be made to protect the data.

18 Funding

Administration and analysis will be carried out by an employee funded for one year by Siemens Healthineers, Erlangen, Germany. No additional funds are available.

Bibliography

Andronikou, S., Lambert, E., Halton, J., et al. 2017. Guidelines for the use of chest radiographs in community-acquired pneumonia in children and adolescents. *Pediatric Radiology* 47(11), pp. 1405–1411.

Anon 2014. *Radiation Emissions from Computed Tomography: A Review of the Risk of Cancer and Guidelines*. Ottawa (ON): Canadian Agency for Drugs and Technologies in Health.

Berrington de González, A., Mahesh, M., Kim, K.-P., et al. 2009. Projected cancer risks from computed tomographic scans performed in the United States in 2007. *Archives of Internal Medicine* 169(22), pp. 2071–2077.

Braun, F.M., Johnson, T.R.C., Sommer, W.H., Thierfelder, K.M. and Meinel, F.G. 2015. Chest CT using spectral filtration: radiation dose, image quality, and spectrum of clinical utility. *European Radiology* 25(6), pp. 1598–1606.

Brenner, D.J. and Hall, E.J. 2007. Computed tomography--an increasing source of radiation exposure. *The New England Journal of Medicine* 357(22), pp. 2277–2284.

Bundesministerium für Arbeit, Soziales, Gesundheit und Konsumentenschutz 2018a. Strahlenanwendung in der Medizin | Gesundheitsportal [Online]. Available at: https://www.gesundheit.gv.at/leben/umwelt/radiokativitaet/medizin [Accessed: 22 November 2018].

Bundesministerium für Arbeit, Soziales, Gesundheit und Konsumentenschutz 2018b. Strahlenbelastung in Österreich | Gesundheitsportal [Online]. Available at: https://www.gesundheit.gv.at/leben/umwelt/radiokativitaet/strahlenbelastung [Accessed: 22 November 2018].

Cardinale, L., Priola, A.M., Moretti, F. and Volpicelli, G. 2014. Effectiveness of chest radiography, lung ultrasound and thoracic computed tomography in the diagnosis of congestive heart failure. *World journal of radiology* 6(6), pp. 230–237.

Chalmers, J.D. 2016. The Modern Diagnostic Approach to Community-Acquired Pneumonia in Adults. *Seminars in Respiratory and Critical Care Medicine* 37(6), pp. 876–885.

Chawla, A., Rajendran, S., Yung, W.H., Babu, S.B. and Peh, W.C. 2016. Chest radiography in acute aortic syndrome: pearls and pitfalls. *Emergency Radiology* 23(4), pp. 405–412.

Ditto, M., Stangl, K., Landstetter, C., Korner, M. and Dauke, M. 2013. *AGES Radioaktivität und Strahlung in Österreich 2012.pdf*. Bundesministerium für Gesundheit.

Fintelmann, F.J., Gottumukkala, R.V., McDermott, S., Gilman, M.D., Lennes, I.T. and Shepard, J.-A.O. 2017. Lung cancer screening: why, when, and how? *Radiologic Clinics of North America* 55(6), pp. 1163–1181.

Haubenreisser, H., Meyer, M., Sudarski, S., Allmendinger, T., Schoenberg, S.O. and Henzler, T. 2015. Unenhanced third-generation dual-source chest CT using a tin filter for spectral shaping at 100kVp. *European Journal of Radiology* 84(8), pp. 1608–1613.

Horeweg, N., Scholten, E.T., de Jong, P.A., et al. 2014. Detection of lung cancer through low-dose CT screening (NELSON): a prespecified analysis of screening test performance and interval cancers. *The Lancet Oncology* 15(12), pp. 1342–1350.

Kim, Y., Kim, Y.K., Lee, B.E., et al. 2015. Ultra-Low-Dose CT of the Thorax Using Iterative Reconstruction: Evaluation of Image Quality and Radiation Dose Reduction. *American Journal of Roentgenology* 204(6), pp. 1197–1202.

Kubo, T., Ohno, Y., Kauczor, H.U. and Hatabu, H. 2014. Radiation dose reduction in chest CT--review of available options. *European Journal of Radiology* 83(10), pp. 1953–1961.

Kubo, T., Ohno, Y., Seo, J.B., et al. 2017. Securing safe and informative thoracic CT examinations-Progress of radiation dose reduction techniques. *European Journal of Radiology* 86, pp. 313–319.

Long, B., Long, D. and Koyfman, A. 2017. Emergency Medicine Evaluation of Community-Acquired Pneumonia: History, Examination, Imaging and Laboratory Assessment, and Risk Scores. *The Journal of Emergency Medicine* 53(5), pp. 642–652.

Macri, F., Greffier, J., Pereira, F., et al. 2016. Value of ultra-low-dose chest CT with iterative reconstruction for selected emergency room patients with acute dyspnea. *European Journal of Radiology* 85(9), pp. 1637–1644.

Martindale, J.L., Wakai, A., Collins, S.P., et al. 2016. Diagnosing Acute Heart Failure in the Emergency Department: A Systematic Review and Meta-analysis. *Academic Emergency Medicine* 23(3), pp. 223–242.

Martini, K., Barth, B.K., Nguyen-Kim, T.D.L., Baumueller, S., Alkadhi, H. and Frauenfelder, T. 2016. Evaluation of pulmonary nodules and infection on chest CT with radiation dose equivalent

to chest radiography: Prospective intra-individual comparison study to standard dose CT. *European Journal of Radiology* 85(2), pp. 360–365.

Messerli, M., Giannopoulos, A.A., Leschka, S., et al. 2017. Diagnostic accuracy of chest X-ray dose-equivalent CT for assessing calcified atherosclerotic burden of the thoracic aorta. *The British Journal of Radiology* 90(1080), p. 20170469.

Messerli, M., Hechelhammer, L., Leschka, S., Warschkow, R., Wildermuth, S. and Bauer, R.W. 2017. Coronary risk assessment at X-ray dose equivalent ungated chest CT: Results of a multi-reader study. *Clinical Imaging* 49(0), pp. 73–79.

Messerli, M., Ottilinger, T., Warschkow, R., et al. 2017. Emphysema quantification and lung volumetry in chest X-ray equivalent ultralow dose CT - Intra-individual comparison with standard dose CT. *European Journal of Radiology* 91, pp. 1–9.

Moore, C.L., Daniels, B., Ghita, M., et al. 2015. Accuracy of reduced-dose computed tomography for ureteral stones in emergency department patients. *Annals of Emergency Medicine* 65(2), pp. 189–98.e2.

Moser, J.B., Sheard, S.L., Edyvean, S. and Vlahos, I. 2017. Radiation dose-reduction strategies in thoracic CT. *Clinical Radiology* 72(5), pp. 407–420.

National Lung Screening Trial Research Team, Aberle, D.R., Adams, A.M., et al. 2011. Reduced lung-cancer mortality with low-dose computed tomographic screening. *The New England Journal of Medicine* 365(5), pp. 395–409.

O'Hora, L. and Foley, S.J. 2018. Iterative reconstruction and automatic tube voltage selection reduce clinical CT radiation doses and image noise. *Radiography (London, England : 1995)* 24(1), pp. 28–32.

Rob, S., Bryant, T., Wilson, I. and Somani, B.K. 2017. Ultra-low-dose, low-dose, and standard-dose CT of the kidney, ureters, and bladder: is there a difference? Results from a systematic review of the literature. *Clinical Radiology* 72(1), pp. 11–15.

Ruchalski, K.L. and Brown, K. 2016. Lung cancer screening update. *Journal of Thoracic Imaging* 31(4), pp. 190–200.

Schegerer, A.A. 2016. *Bekanntmachung der aktualisierten diagnostischen Referenzwerte für diagnostische und interventionelle Röntgenanwendungen*. Bundesministerium der Justiz und für Verbraucherschutz Deutschland.

Suntharalingam, S., Allmendinger, T., Blex, S., et al. 2018. Spectral beam shaping in unenhanced chest CT examinations: A phantom study on dose reduction and image quality. *Academic Radiology* 25(2), pp. 153–158.
van der Aalst, C.M., Ten Haaf, K. and de Koning, H.J. 2016. Lung cancer screening: latest developments and unanswered questions. *The Lancet. Respiratory medicine* 4(9), pp. 749–761.
Vardhanabhuti, V., Pang, C.-L., Tenant, S., Taylor, J., Hyde, C. and Roobottom, C. 2017. Prospective intra-individual comparison of standard dose versus reduced-dose thoracic CT using hybrid and pure iterative reconstruction in a follow-up cohort of pulmonary nodules-Effect of detectability of pulmonary nodules with lowering dose based on nodule size, type and body mass index. *European Journal of Radiology* 91, pp. 130–141.
Wachabauer, D. and Röthlin, F. 2017. *Aktualisierung der diagnostischen Referenzwerte für Österreich*. Gesundheit Österreich GmbH.
Walter, J.E., Heuvelmans, M.A., de Jong, P.A., et al. 2016. Occurrence and lung cancer probability of new solid nodules at incidence screening with low-dose CT: analysis of data from the randomised, controlled NELSON trial. *The Lancet Oncology* 17(7), pp. 907–916.
Yousaf-Khan, U., van der Aalst, C., de Jong, P.A., Heuvelmans, M., Scholten, E., Lammers, J.-W., et al. 2017. Final screening round of the NELSON lung cancer screening trial: the effect of a 2.5-year screening interval. *Thorax* 72(1), pp. 48–56.
Yousaf-Khan, U., van der Aalst, C., de Jong, P.A., Heuvelmans, M., Scholten, E., Walter, J., et al. 2017. Risk stratification based on screening history: the NELSON lung cancer screening study. *Thorax* 72(9), pp. 819–824.
Zinsser, D., Marcus, R., Othman, A.E., et al. 2018. Dose Reduction and Dose Management in Computed Tomography - State of the Art. *RoFo: Fortschritte Auf Dem Gebiete der Rontgenstrahlen und der Nuklearmedizin* 190(6), pp. 531–541.
