# Supplementary figures and images for: Ultra-low-dose CT vs. chest X-ray in non-traumatic emergency department patients – a prospective randomised crossover cohort trial
Source: eClinicalMedicine. 2023 Oct 17;65:102267. doi: 10.1016/j.eclinm.2023.102267 (PMC10590727; doi:10.1016/j.eclinm.2023.102267)

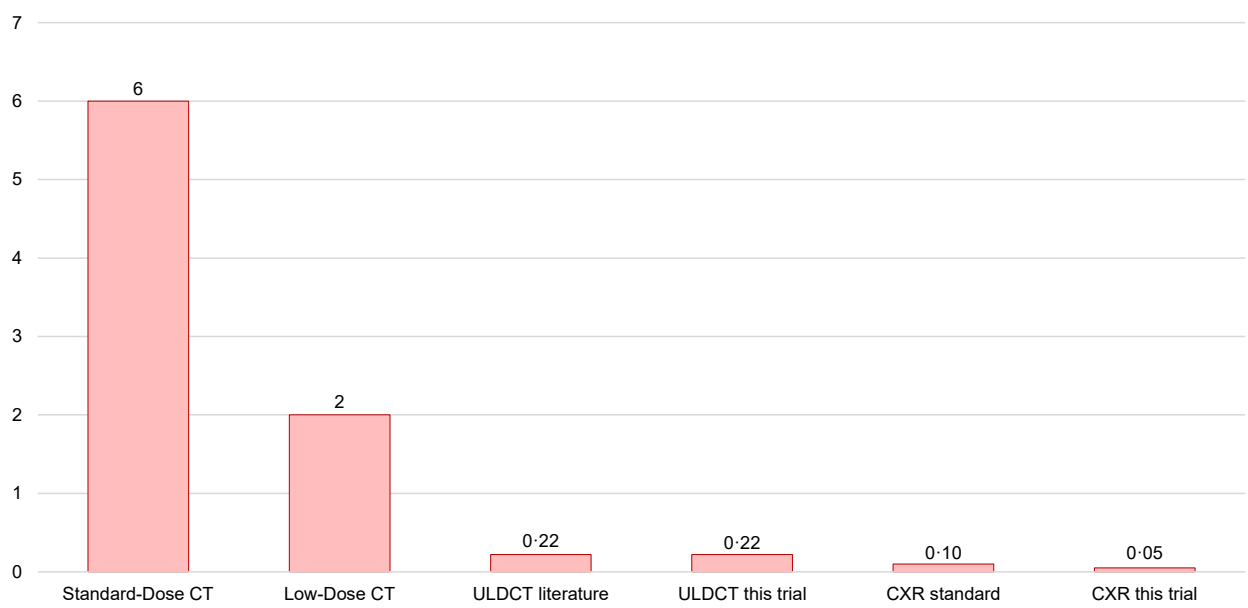

Supplement: Figure E1 [file mmc3.pdf]
